# Construction of human pluripotent stem cell-derived testicular organoids and their use as humanized testis models for evaluating the effects of semaglutide

**DOI:** 10.7150/thno.104523

**Published:** 2025-01-27

**Authors:** Rufei Huang, Huan Xia, Tao Meng, Yufei Fan, Xun Tang, Yifang Li, Tiantian Zhang, Jingxian Deng, Bing Yao, Yadong Huang, Yan Yang

**Affiliations:** 1 State Key Laboratory of Bioactive Molecules and Druggability Assessment, Guangdong Basic Research Center of Excellence for Natural Bioactive Molecules and Discovery of Innovative Drugs, College of Life Science and Technology, Jinan University, Guangzhou, 510632, China.; 2 Department of Reproductive Medicine, Jinling Hospital, Affiliated Hospital of Medical School, Nanjing University, Nanjing, 210002, China.; 3 National Engineering Research Center of Genetic Medicine, Guangzhou, 510632, China.; 4 Guangdong Province Key Laboratory of Bioengineering Medicine, Guangzhou, 510632, China.

**Keywords:** human induced pluripotent stem cells, differentiation, testicular organoids, GLP-1 receptor agonist, semaglutide

## Abstract

**Background:** The generation of human testicular organoids from human induced pluripotent stem cells (hiPSCs) presents exciting opportunities for gonadal developmental biology, and reproductive disease modeling. However, creating organoids that closely mimic the tissue structure of testes remains challenging.

**Methods:** In this study, we established a method for generating testicular organoids (TOs) from hiPSCs using a stepwise differentiation approach and a combination of hanging drop and rotational culture systems. The capability of hiPSC-derived precursor testicular cells to self-assemble into organoids was confirmed by detection of morphology, single-cell RNA-sequencing, and protein profiles. The reliability of testicular organoids as a drug evaluation model was assessed by the measurements of transcriptome signatures and functional features, including hormone responsiveness and blood-testis barrier (BTB) formation, and drug sensitivity assessment by recording cell viability and BTB integrity in organoids exposed to reproductive toxicants. Finally, we applied testicular organoids to evaluate the effects of semaglutide, a glucagon-like peptide-1 receptor agonist (GLP-1 RA), on testicular function, thereby underscoring their utility as a model for drug evaluation.

**Results:** These organoids exhibited testicular cord-like structures and BTB function. RNA sequencing and functional assays confirmed that testicular organoids possess gene expression profiles and endocrine functions regulated by gonadotropins, closely resembling those of testicular tissue. Notably, these organoids displayed sensitivity to semaglutide. Treatment with semaglutide resulted in reduced testosterone levels and downregulation of *INHBB* expression, aligning with previous clinical observations.

**Conclusions:** These findings introduced a method for generating testicular organoids from human pluripotent stem cells, highlighting their potential as valuable models for studying testicular function, drug toxicity, and the effects of compounds like semaglutide on testicular health.

## Introduction

The emergence of organoid technology has revolutionized regenerative medicine, disease modeling, and drug screening 1-3. These organoids are three-dimensional (3D) cell cultures derived from primary tissues or stem cells, characterized by their complex 3D structure, which enables them to replicate the intricate structure and function of human organs 4, 5. And this technology empowers scientists to investigate *in vivo* organ development, elucidate human disease mechanisms, and assess therapeutic responses within a more physiologically relevant context, offering greater precision than traditional 2D cell cultures 6.

In the context of the testis, the utility of organoids is particularly pronounced, given the organ's reliance on intricate intercellular interactions and hormonal responses 7, 8. Specifically, the testis is composed of multiple cell types, including Sertoli cells (SCs), Leydig cells (LCs), peritubular myoid cells (PTMs), and germ cells (GCs) 9. SCs secrete lactate and inhibin B (INHBB) under the regulation of follicle-stimulating hormone (FSH). Lactate serves as the optimal energy source for germ cells, while INHBB is crucial for the proliferation and differentiation of spermatogonial stem cells (SSCs) 10, 11. Additionally, SCs produce anti-Müllerian hormone (AMH), which is instrumental in the regression of female reproductive structures during male development 12. Luteinizing hormone (LH) induces LCs to secrete testosterone, which binds to androgen receptor (AR) in SCs, thereby regulating the integrity of the blood-testis barrier (BTB), the development of spermatids, and the release of sperm 13. The presence of specific receptors in various cell types in the testis, along with their capacity to respond to hormonal signals, is essential for maintaining testicular function and male fertility 14, 15. Therefore, models that accurately simulate testicular cell composition and hormonal responses are invaluable for research in reproductive medicine and toxicology.

Testicular organoids are a promising model for investigating male reproductive health and evaluating drug toxicity. These organoids can effectively replicate the intercellular interactions and complex microenvironment of the testis, providing a more comprehensive perspective for understanding how drugs influence testicular function 16-18. The impact of glucagon-like peptide-1 receptor agonists (GLP-1 RAs), such as semaglutide, on male reproduction, a class of drugs approved for weight loss, is a case in point. While they show promise for obesity treatment, their potential effects on reproductive health are contentious and require nuanced investigation 19-21. Current research primarily relies on mouse cell lines and rodent models, with a notable lack of humanized models to conduct more accurate studies 22, 23. The humanized testicular organoid models are scarce due to the limited number of human primary testicular cells, their restricted expansion in culture, and the ethical and individual variances associated with their acquisition. Furthermore, the cells used are sourced from fully developed human testes, which lack the stem cells essential for tissue morphogenesis, resulting in organoids with incomplete tubular structures, limited self-organization ability and secretory function 18, 24.

Human pluripotent stem cells (hPSCs), including human embryonic stem cells (hESCs) and human induced pluripotent stem cells (hiPSCs), offer a viable solution due to their ability to differentiate into testicular cells. These cells also possess self-renewal characteristics and ensure a consistent cell source, making them ideal candidates for organoid technology 25. Although stem cell differentiation into various organoids has been explored, research specifically focused on differentiating stem cells into testicular cells and forming functional testicular organoids remains limited 26. Sepponen *et al.* guided hESCs through intermediate mesoderm (IM) that ultimately generated the bipotential gonadal cells in monolayer cultures and analyzed the role of sequential inhibition and activation of BMP signaling during induction of IM 27. Their team further demonstrated the successful reprogramming of hiPSCs into male gonadal-like cells responsive to FSH by combining directed differentiation of hiPSCs with the activation of endogenous NR5A1 expression 28. In a separate study, Knarston *et al.* utilized growth factors and compounds to sequentially activate WNT and BMP signaling in hiPSCs, leading to the expression of bipotential gonadal markers without the need for co-culture or transfection. They demonstrated that the 3D culture of these cells resulted in well-defined tissue architecture and unique expression of SCs markers 29. However, functional similarities to native gonadal tissue, such as the BTB and androgen production, have not been conclusively established in these iPSC-derived organoids.

In this study, we developed a method to generate testicular organoids from hiPSCs, leveraging small molecule compounds to induce differentiation and a combination of hanging drop and rotational culture systems to assemble these cells into organoids. Our organoids closely resemble human testicular tissue, exhibiting a cell aggregation pattern with certain tubule-like characteristics, as well as hormone responsiveness and tight junction function. Furthermore, we applied these organoids to assess the impact of semaglutide on testicular function, providing a more physiologically relevant platform for investigating the reproductive health implications of obesity treatments. This work not only addresses the limitations of existing models but also provides the potential for establishing *in vitro* male reproductive disease models.

## Materials and Methods

### Cell culture and monolayer differentiation

ATCC-DYR0100 male human induced pluripotent stem cells (iPSCs) (ACS-1011^TM^) were obtained from the American Type Culture Collection (USA). iPSCs were seeded on dishes coated with Matrigel (Cat# 354277, Corning, Tewksbury, MA, USA) and maintained in Essential 8^TM^ medium (E8, Cat# A1517001, Thermo Fisher Scientific, Waltham, MA, USA). The E8 medium was changed daily. For differentiation, cells were plated on Matrigel-coated plates at a density of 10,000 cells per square centimeter, and 10 µM of a ROCK inhibitor (Y-27632, Cat# S1049, Selleckchem, Houston, TX, USA) was added to E8 medium. At day 0, the medium was changed to Essential 6^TM^ medium (E6, Cat# A1516401, Thermo Fisher Scientific) supplemented with 10% [v/v] KnockOut^TM^ Serum Replacement (KSR, Cat# 10828028, Thermo Fisher Scientific), 3 µM CHIR-99021 (CHIR, Cat# S1263, Selleckchem), and 100 ng/mL activin A (Cat# HY-P70311, MedChemExpress, Monmouth Junction, NJ, USA). On day 2, the medium was replaced with E6 medium containing only 3 µM CHIR and cultured for 2 days. Subsequently, in the next 3 days, 200 ng/mL FGF9 (Cat# HY-P73053, MedChemExpress), 10 ng/mL BMP4 (Cat# HY-P7007, MedChemExpress), and 1 µg/mL heparin (Cat# S1346, Selleckchem) were added to the differentiation medium. The conditioned medium was changed daily.

### Organoid preparation and culture

On the 7th day of cell differentiation, cells were dissociated with 0.25% trypsin-EDTA, and then aggregated into organoids by hanging drop culture as described previously 30. Briefly, the dissociated cells were resuspended in DMEM medium, counted, and then inoculated onto the inside of a sterile square dish lid at densities of 1000, 3000, and 5000 cells per drop (20 µL) of medium using a multi-channel pipette. The hanging drop conditional medium was supplemented with 20% [v/v] KSR, 0.5% [v/v] Matrigel Matrix (Cat# 354262, Corning), 200 ng/mL FGF9, 10 ng/mL BMP4, and 1 µg/mL heparin. The hanging drops were cultured at 34 °C and 5% CO_2_ for 1, 3, or 5 days. Subsequently, the cell spheroids were collected into a 125 mL polycarbonate Erlenmeyer flask with vent cap (Cat# 431143, Corning). The Erlenmeyer flask contained 15 mL DMEM medium, which is supplemented with 20% [v/v] KSR, 50 ng/mL EGF (Cat# HY-P7109, MedChemExpress), 10 ng/mL FSH (Cat# HY-P70237, MedChemExpress), 10 ng/mL LH (Cat# HOR-261, ProSpec), 50 ng/mL IGF (Cat# HY-P1777, MedChemExpress), 1 µM Retinoic acid (Cat# S1653, Selleckchem), 10 ng/mL BMP4, 50 ng/mL FGF9, and 1 µg/mL heparin. The Erlenmeyer flask was transferred to a 34 °C constant-temperature culture shaker at 120 to 140 rpm for rotation culture. The culture medium was replaced every five days.

### Morphological analysis and Hematoxylin and Eosin (H&E) staining

To statistically analyze the areas of cell spheroids, we captured their images using an optical microscope (Nikon, Tokyo, Japan) at predetermined time intervals. We then utilized ImageJ software (version 1.48) to quantify and calculate the average areas from 50 randomly selected images. For histological analysis, the cell spheroids were embedded in 3% agarose and fixed with 4% paraformaldehyde overnight at 4 °C. The agarose blocks containing the organoids were then removed and dehydrated in graded ethanol (70%, 80%, 90%, 95%, and 100%). The dehydrated agarose blocks were treated with xylene twice for 5 min each time, then transferred to a tissue embedding box and embedded in paraffin. The paraffin blocks were cut in their entirety into 5 µm thick sections, deparaffinized and stained with hematoxylin and eosin (H&E). After final dehydration through graded ethanol and soaking in xylene for 10 min, the slides were mounted using neutral gum. All H&E images were taken using an optical microscope (Nikon).

### RNA extraction and qRT-PCR

Total RNA was extracted from monolayer cells of differentiation at days 0 and 7, and from organoids at different culture times using TRIzol reagent (Cat# 15596018, Invitrogen, Carlsbad, CA, USA) according to the manufacturer's protocol. One microgram of total RNA was reverse transcribed into cDNA using PrimeScript™ RT Master Mix (Cat# RR036A, TaKaRa, Osaka, Japan). The cDNA template was diluted 1:2, and 2 µL of the diluted template was used for 20 µL of the qRT-PCR assay using ChamQ SYBR qPCR Master Mix (Cat# Q311-02, Vazyme Biotech, Nanjing, China). The CFX Connect Real-Time PCR Detection System (Bio-Rad Laboratories, Hercules, CA, USA) and the CFX Manager Software (version 3.0) were used to visualize the PCR data. Quantification was performed via the comparative 2^-ΔΔCt^ method. Data were normalized to the house-keeping gene *GAPDH* and quantified relative to undifferentiated hiPSCs. The primer sequences used are listed in Table S1.

### RNA sequencing analyses

Human testicular tissue was obtained from obstructive azoospermia male donors (aged 29 years, n = 1). Samples were obtained through Nanjing University Hospital with informed written consent from the patients (ethical approval 2015NZKY-017-02). As described previously, total RNA was extracted from undifferentiated hiPSCs, organoids at days 3 and 8, and human testicular tissue using TRIzol reagent. RNA quality monitoring, library preparation, and sequencing were performed by the high-throughput laboratory of Wuhan BGI Technology Services Co., Ltd. The A260/A280, A260/A230 and RIN values of the sample RNA were detected by the Fragment Analyzer to confirm the RNA quality and integrity. Synthesis and amplification of cDNAs using 200 ng of purified total RNA, and construction of single-stranded circular cDNA libraries for RNA sequencing were performed. Single-stranded circular DNA molecules were replicated through rolling cycle amplification to form a DNA nanoball (DNB) containing multiple copies. The DNBs were added to the patterned nanoarrays using high-intensity DNA nanochip technique, and sequenced through combinatorial Probe-Anchor Synthesis technology (cPAS).

### Sequencing data analysis

The raw data obtained from sequencing was quality controlled (QC) and filtered using SOAPnuke (version 1.5.6). Briefly, clean data was obtained by excluding reads that contained sequencing adapter, had an unknown base N content exceeding 5%, or exhibited a low-quality base ratio greater than 20%. Subsequently, the clean data were aligned with the human genome (GCF_000001405.39_GRCh38.p13) using HISAT2 software (version 2.1.0), and the alignment results were subjected to the second quality control (QC of alignment). RSEM software (version 1.3.1) was used for gene expression quantification, and pheatmap (version 1.0.8) was used to draw clustering heatmaps of gene expression levels in different samples. Differentially expressed genes (DEGs) were identified using DESeq2 (version 1.4.5), with a Q value threshold of ≤ 0.05. Furthermore, GO and KEGG enrichment analyses on the differentially expressed genes were conducted using Phyper based on the hypergeometric test, applying a threshold of Q value ≤ 0.05.

### Organoid staining and confocal analysis

Testicular organoids for confocal analysis were collected and fixed with 4% paraformaldehyde at room temperature for 30 min. The organoids were permeabilized in PBS containing 0.5% Triton X-100 for 30 min and then blocked with blocking buffer (Cat# P0102, Beyotime, Shanghai, China) for 1 h with rocking on a shaker at 60-80 rpm to block the non-specific adhesion sites. The supernatant was then removed, and the organoids were incubated with primary antibodies overnight with rocking at 4 °C, followed by the relevant secondary antibodies for 2 h rocking at room temperature. DAPI (Cat# AR1177, Bosterbio, Pleasanton, CA, USA) was added and the organoids were incubated for a further 30 min. After each experimental step such as fixation, permeabilization, primary and secondary antibody incubation, the organoids need to be washed in PBS at least 3 times for 5 min each time. The organoids were transferred with minimal PBS onto a glass slide, followed by drops of anti-fluorescence decay mounting medium (Cat# AR1109, Bosterbio). The organoids were then analyzed using an LSM900 confocal microscope (Zeiss, Jena, Germany). Details of the primary and secondary antibodies are listed in Table S2.

### Single-cell RNA-sequencing and data analysis

The testicular organoids were dissociated to single cells by the treatment of 0.25% trypsin-EDTA for 10 min, and washed with 1×PBS. Trypan blue staining was used to quantify the cells and assess their viability. Then, droplet-based single-cell RNA-seq library preparation was conducted using the 10X Genomics Chromium platform. In brief, the prepared single-cell suspension was loaded onto a microfluidic chip, and cells and gel beads with cell barcodes were encapsulated in droplets. Within each droplet, the cells were lysed, allowing the mRNA to bind to the cell barcodes on the beads, resulting in the formation of Single Cell GEMs (Gel Bead-in-Emulsions). Inside the droplet, the mRNA underwent reverse transcription to generate cDNA. The cDNA products were fragmented, end-repaired, and base A was added to the 3' end of each chain. Finally, adapter ligation and PCR amplification were performed to facilitate sample indexing and the construction of the 3′ RNA-Seq library. After quality control of the library, sequencing was conducted on the Illumina NextSeq 500 system. The raw off-machine FASTQ data were analyzed using Cell Ranger (version 7.2.0), and the RNA reads were aligned to the GRCh38 human genome using STAR software. A total of 12,658 cells were captured in the organoid single-cell suspension, with a median of 4358 genes detected per cell and a median of 11,334 UMIs detected per cell. For cell quality control, we utilized the Seurat package (version 3.0.2) to filter out cells based on the following criteria: 1) fewer than 200 genes or more than 90% of the maximum number of genes; 2) a mitochondrial gene ratio greater than 15%; 3) identification and removal of potential doublets using DoubletDetection; and 4) after correcting for the effects of the cell cycle, the final number of cells obtained was 10,053. For cell clustering, PCA analysis (n = 15) was performed using 2000 highly variable genes in the dataset, followed by further dimension reduction and clustering using uniform manifold approximation projection (UMAP) analysis. The FindAllMarkers function in the Seurat package (log fold-change > 0.25, and Padj ≤ 0.05) was employed to identify differentially expressed genes for each cell cluster, which were subsequently categorized into major cell types based on the expression of typical marker genes. Monocle2 (version 2.10.1) was used for pseudo-time trajectory analysis to determine the trajectory of pseudo-time differentiation and development of cells. Additionally, we integrated testicular organoid scRNA-seq data with human testis scRNA-seq data from three embryonic and fetal stages (6, 8, and 16 weeks postfertilization) available in GSE143356 31. Clustering analysis was then performed using the Seurat multiple dataset integration method and visualized using UMAP analysis.

### Testosterone and lactate measurements

To examine the hormone secretion and energy metabolism capabilities of testicular organoids, we measured testosterone levels and lactate production after LH or FSH treatment. Briefly, the organoids at day 8 were treated with the medium containing either LH or FSH at concentrations of 1, 10, and 100 ng/mL for a duration of 24 h. Conditioned medium collected from organoids was vortexed and centrifuged prior to its use in testosterone and lactate assays. The concentration of testosterone in the supernatant was determined using an Iodine[^125^I] Testosterone Radioimmunoassay Kit (Cat# B10B, Northern Institute of Biotechnology, Beijing, China). The lactate concentration was measured using a commercial lactate assay kit (Cat# A019-2-1, Nanjing Jiancheng Bioengineering Institute, Nanjing, China). Reagents for each kit were prepared according to the manufacturer's instructions. Basal medium for organoid culture (DMEM containing 10% KSR) was used as a blank in the assay.

### Western Blotting

Organoids were collected, and protein samples were obtained by lysis with RIPA buffer (Cat# 89900, Thermo Fisher Scientific) containing a protease inhibitor cocktail (Cat# 20-116, Millipore, St. Louis, MO, USA). Protein concentrations in the samples were determined using a BCA Protein Assay Kit (Cat# 23225, Thermo Fisher Scientific) and then normalized across all samples. Each sample containing 35 µg of protein was separated by 10% SDS-PAGE gel, and the proteins were transferred to a PVDF membrane (Cat# IPVH00010, Millipore) using an electroblotter (Bio-Rad, Hercules, CA, USA). After blocking with 5% skim milk for 1 h, polyclonal rabbit anti-FSHR, polyclonal rabbit anti-LHCGR, polyclonal rabbit anti-AR, and polyclonal rabbit anti-GLP-1R were added and incubated overnight at 4 °C. The membranes were washed five times with TBST (7 min each time) and incubated with horseradish peroxidase (HRP)-conjugated secondary antibody for 1 h at room temperature. Membranes were then rinsed 5 times (7 min each time) with TBST, and immunoreactions were detected by enhanced chemiluminescence (ECL) detection. The protein expression was normalized to GAPDH. Details of the primary antibodies are listed in Table S2.

### Evaluation of blood-testis barrier (BTB) integrity in organoids

EZ-Link^TM^ Sulfo-N-hydroxysuccinimide (NHS)-LC-Biotin (Cat# 21335, Thermo Fisher Scientific), a water-soluble and membrane-impermeable biotinylation reagent, was used to assess the permeability of BTB in organoids. Briefly, day 8 organoids were pretreated with 10 µM CdCl_2_ (Cat# 202908, Sigma-Aldrich, St. Louis, MO, USA), etoposide (Cat# S1225, Selleckchem), and doxorubicin (Cat# E2516, Selleckchem) for 24 h and then incubated with 10 mg/mL biotin solution for 10 min at 34 °C, 5% CO_2_. Also, the control organoids were treated with corresponding solvents and incubated under the same conditions to serve as a baseline control. The organoids were washed twice with PBS and then fixed with 4% paraformaldehyde for 30 min at room temperature. After washing three times with PBS and blocking with 5% BSA for 1 h, the organoids were incubated with Alexa Fluor 568-labeled streptavidin (diluted 1:500 in 1% BSA) for 2 h at room temperature. The organoids were then washed three times with PBS and treated with DAPI for an additional 30 min. The organoids were examined for staining using an LSM900 confocal microscope (Zeiss).

### Drug target mendelian randomization (MR)

The drug target MR method was used to explore the causal relationship between GLP-1 RAs, which are FDA-approved for regulating blood glucose and body weight, and male reproductive diseases alongside sex hormone levels. For the selection of genetic tools, the cis-expression quantitative trait locus (cis-eQTL) of the target gene GLP-1R of GLP-1 RAs was utilized as the instrumental variable (IV). Following the methodology outlined by Sun *et al.* 32, 22 eQTL single nucleotide polymorphisms (SNPs) significantly associated with GLP-1R expression in blood were selected as genetic tools (Table S3). To assess the strength of the IVs related to the exposure trait, we calculated the F statistic (F = beta^2^/se^2^) for each SNP, ensuring that the F value for each SNP exceeded 10. In addition, the association of the genetic tool with type 2 diabetes, body mass index (BMI), and glycated hemoglobin (HbA1c) was evaluated as alternatives to exposure to GLP1-RAs. For the source of outcome data, all outcome data populations were restricted to European populations to ensure the strength of MR analysis. We obtained genome-wide association study (GWAS) data for orchitis and epididymitis, a common male reproductive disease, from the R9 release of the FinnGen consortium 33. FSH, LH, and free testosterone level data were sourced from the GWAS Catalog database 34, while data on INHBB and bioavailable testosterone were acquired from the IEU Open GWAS project 35. Detailed information for each outcome data is given in Table S4. For statistical analysis, the random effects inverse variance weighted (IVW) method was first employed to calculate the association odds ratio (OR) and corresponding confidence interval (CI) between cis-eQTL and outcome, thereby assessing the impact of IVs on the outcomes. Furthermore, the median weighted method was additionally used for effect analysis to improve the robustness of the results. For sensitivity analysis, Cochrane Q statistics and MR-Egger method were used to assess potential directional pleiotropy in MR analysis. A p-value of less than 0.05 indicated the presence of pleiotropy in the direction assessed. All analyses were conducted using R software (version 4.3.0) with the R packages TwoSampleMR (version 0.5.7) and MendelianRandomization (version 0.9.0) for MR analysis. All included GWAS studies received approval from the relevant institutional review boards. Since this study involved a secondary analysis of publicly available data, no additional ethical approval was required.

### Network pharmacology and bioinformatics analysis

To elucidate the molecular mechanisms underlying the differentiation of human pluripotent stem cells into testicular organoids, network pharmacology and bioinformatics analyses were employed. First, the targets of five reprogramming compounds—CHIR, activin A, BMP4, FGF9, and heparin—were predicted and screened. The targets of CHIR, activin A, and heparin were searched using the SwissTargetPrediction 36, Therapeutic Target Database (TTD) 37, DrugBank 38, ChEMBL 39, and STITCH databases 40. Proteins that interact with the cytokines BMP4 and FGF9 were predicted via the STRING database 41, applying a threshold for interaction scores of ≥ 0.7. Subsequently, the target and protein names were converted into gene names using the UniProt database 42. These target genes were then cross-collected with the DEGs between hiPSCs and day 3 organoids (|log_2_FoldChange| ≥ 2, Q value ≤ 0.01) using the Venny 2.1.0 analysis tool to form a Venny diagram, thereby identifying potential targets for compound-induced testicular organoid formation. The interactions among potential targets were obtained through the STRING database, and the minimum combined score was set to 0.4. The protein-protein interaction (PPI) network was constructed using Cytoscape 3.7.2 software. The core targets were further screened according to the degree value and betweenness centrality, with the conditions of degree value > 35 and betweenness centrality > 0.024. Finally, Gene Ontology (GO) and Kyoto Encyclopedia of Genes and Genomes (KEGG) enrichment analysis were performed to explore the potential molecular mechanism of reprogramming compounds' induction of testicular organoids.

### Statistical analysis

All experiments were performed in triplicate, and data are expressed as mean ± SD. Statistical analysis was performed using GraphPad Prism software (version 8.0, San Diego, CA, USA). Statistical significance for more than two groups was analyzed by a two-tailed Student's t-test or one-way analysis of variance (ANOVA), where *P* < 0.05 was considered minimally significant.

## Results

### Stepwise induction of human precursor testis-like cells from hiPSCs

**Figure 1:**
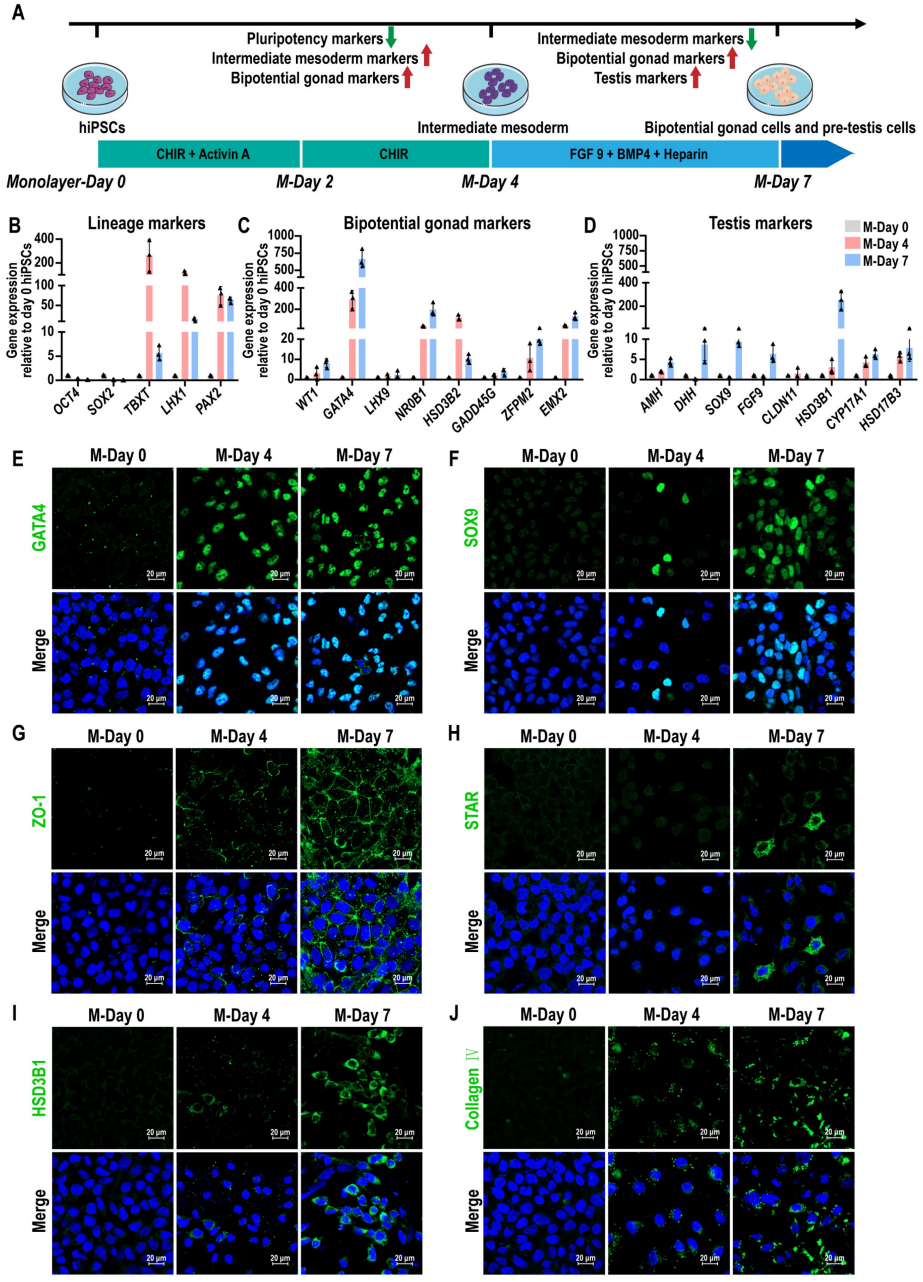
** Conversion of hiPSCs into precursor testis-like cells by stepwise addition of small molecules. (A)** Schematic illustration of the strategy to convert hiPSCs into precursor testis-like cells by using small molecules. **(B-D)** qRT-PCR data of relative gene expression after 4 and 7 days of monolayer differentiation for (B) lineage markers (*OCT4* and *SOX2*, pluripotency; *TBXT*, PS; *LHX1* and *PAX2*, IM), (C) bipotential gonad markers (*WT1*, *GATA4*, *LHX9*, *NR0B1*, *HSD3B2*, *GADD45G*, *ZFPM2*, and *EMX2*), and (D) Testis markers (*AMH*, *DHH*, *SOX9*, *FGF9*,* CLDN11*, *HSD3B1*, *CYP17A1*, and *HSD17B3*). The mRNA copy number of each gene was normalized with *GAPDH*. Gene expression was quantified relative to day 0 hiPSCs (mean ± SD, n = 3 independent experiments). **(E-J)** Immunofluorescence analysis was performed at days 0, 4, and 7 of monolayer differentiation. Bipotential gonad cells: GATA4. Sertoli cells: SOX9 and ZO-1. Leydig cells: STAR and HSD3B1. Basement membrane: Collagen IV. Nuclei were stained with DAPI (blue). Scale bar, 20 µm.

The mammalian gonads originate from the intermediate mesoderm (IM) 43. To generate testicular lineages from hiPSCs, a stepwise directed differentiation approach was employed to guide cells through the IM, ultimately generating bipotent gonad and precursor testicular cells. We developed a protocol for generating precursor testicular cells from hiPSCs by using developmental cues for gonad and testis formation. We induced hiPSCs to differentiate toward IM using activin A and WNT signaling activator CHIR99021 (CHIR). Subsequent treatment with fibroblast growth factor 9 (FGF 9), bone morphogenetic protein 4 (BMP4), and heparin led to an up-regulation of the gonad and precursor testicular cells marker *GATA4* and *SOX9*, and downregulation of pluripotency markers *OCT4* and *SOX2* in the cells (Figure 1A). After FGF9, BMP4, and heparin treatment for 3 days, we observed that hiPSCs, which typically demonstrate adherent colony growth, were induced into epithelial-like cells characterized by distinct intercellular borders. The induced cells primarily exhibited short fusiform or irregular polygonal morphologies (Figure S1A). Pluripotency markers *OCT4* (also known as *POU5F1*) and *SOX2* were most expressed in hiPSCs at day 0 and decreased significantly after treatment, indicating a transition in hiPSCs identity. The expression of posterior primitive streak (PS) markers (*TBXT*) and IM markers (*LHX1* and *PAX2*) reached their peak at day 4, and then decreased at day 7, suggesting that cells passed through the precursor cell population (Figure 1B). The expression levels of several bipotential gonadal markers (*WT1*, *GATA4*, *NR0B1*, *GADD45G*, *ZFPM2*, and *EMX2*), SCs markers (*AMH*, *DHH*, *SOX9*, and *FGF9*), and LCs markers (*HSD3B1*, *CYP17A1*, and *HSD17B3*) were observed to increase gradually between days 4 and 7, indicating that bipotent gonad and testicular cells were induced (Figure 1C-D). However, the expression of *CLDN11*, a marker of the blood-testis barrier (BTB), remained very low. Markers of different cell types were further analyzed by immunofluorescence staining. At day 7, immunofluorescence revealed the presence of GATA4^+^, SOX9^+^, ZO-1^+^, STAR^+^, and HSD3B1^+^ cells, suggesting the emergence of bipotential gonadal cells, Sertoli cells, and Leydig cells, respectively (Figure 1E-I). Additionally, we also observed Collagen IV and fibronectin marked the epithelial basement membrane, with strong expression (Figure 1J, Figure S1B, Figure S9).

These results revealed that bipotential gonad and testis markers were activated when hiPSCs were treated with initial activin A and CHIR induction followed by addition of FGF9, BMP4, and heparin.

### Generate testicular organoids from hiPSC-derived precursor testicular cells

**Figure 2:**
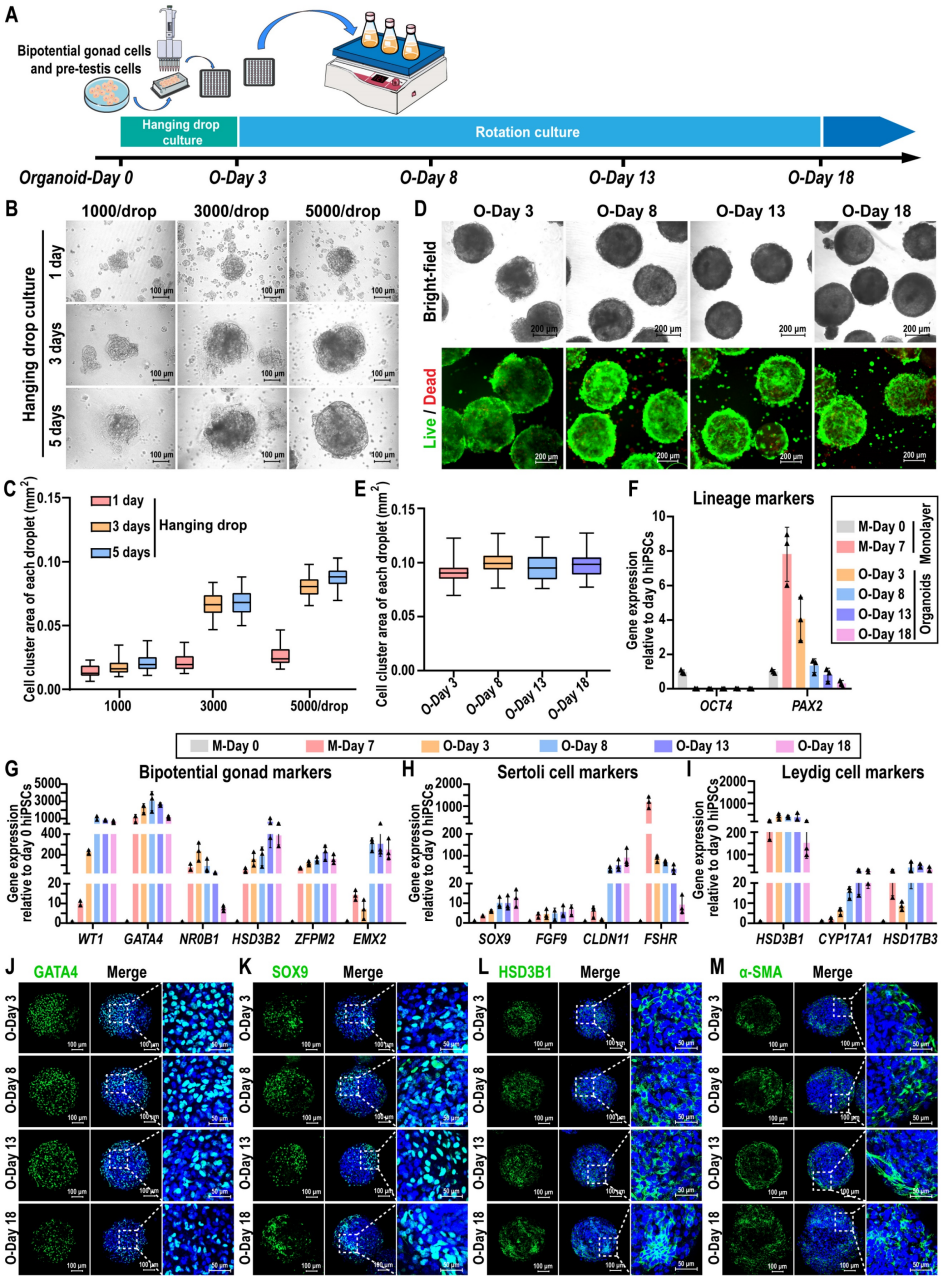
** hiPSC-derived pre-testis cells self-assembled into 3D cell spheroids in an environment that combined hanging drop and rotation culture. (A)** Schematic illustration of the strategy for testicular organoid preparation. The dissociated day 7 monolayer cells were reaggregated and cultured in a hanging drop combined rotation system. **(B-C)** Bright-field images and area statistics of cell spheroids at densities of 1000, 3000, and 5000 cells/drop after 1, 3, and 5 days of hanging drop culture. Image scale bar, 100 µm. The area of cell spheroids was measured by ImageJ (mean ± SD, n = 50). **(D)** Bright-field images and Live/Dead staining images of cell spheroids (5000 cells/drop) after 3, 8, 13, and 18 days of organoid culture. Image scale bar, 200 µm. **(E)** The area of cell spheroids (5000 cells/drop) was measured by ImageJ (mean ± SD, n = 50). **(F-I)** qRT-PCR data of relative gene expression in day 0 and day 7 monolayer cells and day 3, 8, 13, and 18 organoids for (F) lineage markers (*OCT4*, pluripotency; *PAX2*, IM), (G) bipotential gonad markers (*WT1*, *GATA4*, *NR0B1*, *HSD3B2*, *ZFPM2*, and *EMX2*), (H) Sertoli cell markers (*SOX9*, *FGF9*, *CLDN11*, and *FSHR*), and (I) Leydig cell markers (*HSD3B1*, *CYP17A1*, and *HSD17B3*). The mRNA copy number of each gene was normalized with *GAPDH*. Gene expression was quantified relative to day 0 hiPSCs (mean ± SD, n = 3 independent experiments). **(J-M)** Immunofluorescence analysis was conducted on days 3, 8, 13, and 18 organoids. Bipotential gonad cells were identified by the marker GATA4, Sertoli cells by SOX9, Leydig cells by HSD3B1, and peritubular myoid cells by α-SMA. Nuclei were counterstained with DAPI (blue). The image to the right of each merged image shows an enlargement of the white dashed square. Scale bars = 100 µm, and 50 µm in magnified regions.

To create 3D organoids of human testis, we developed a culture system in which hanging drop and rotation culture combined. Briefly, hiPSC-derived precursor testicular cells were dissociated and reaggregated, followed by a 3D culture period of up to 18 days, which included 3 days in hanging drop culture and 15 days in rotation culture (Figure 2A). Cell spheroids were observed using brightfield microscopy on days 1, 3, and 5 following the initiation of hanging drop culture. The results indicated that hiPSC-derived precursor testicular cells aggregated into spheroids at densities of 1000, 3000, and 5000 cells per drop. The spheroids exhibited uniform shapes with clear edges at a density of 5000 cells/drop, while those remained irregular and flat at densities of 1000/drop and 3000/drop until day 5 (Figure 2B). The average area of the spheroids gradually increased from day 1 to 3, with the growth rate beginning to slow down from day 3 to 5 (Figure 2C). Live/dead staining results revealed that no significant cell death was observed in the cell spheroids formed at different cell densities (Figure S2A-C). Therefore, spheroids of 5000 cells/drop were utilized to generate testicular organoids, and the organoids obtained after 3 days of hanging drop culture (O-Day 3) were transferred to an Erlenmeyer flask and cultured rotationally on a horizontal shaker to promote their growth and maturation.

The morphology, gene dynamics, and protein profiles of organoids were examined during rotational culture spanning from O-Day 3 to O-Day 18. These organoids displayed a 3D spherical shape, with distinct and bright edges upon rotation (Figure 2D, Figure S2D). The average area of organoids remained relatively stable throughout the rotational culture (O-Day 3 to O-Day 18) (Figure 2E). Live/dead staining revealed that the organoids maintained high viability on day 18 of culture (Figure 2D), with only a small number of dead cells observed by day 33 (Figure S2E). The key pluripotency gene *OCT4* was only highly expressed in hiPSCs on day 0 of induction, whereas the IM marker *PAX2* reached a peak on day 7 of monolayer differentiation, and declined in the organoids thereafter (Figure 2F, Table S17). Bipotential gonadal markers (*WT1*, *GATA4*, *NR0B1*, *ZFPM2*, and *EMX2*) exhibited high expression in organoids during days 3 to 8, followed by a gradual decrease during rotational culture (Figure 2G, Table S18). In addition to *FSHR*, the expression levels of Sertoli cell markers *SOX9*, *FGF9*, and *CLDN11* in organoids were significantly higher than those observed in day 7 monolayer cells and remained stably high expression throughout the subsequent rotational culture (Figure 2H, Table S19). Notably, *CLDN11* was dramatically induced in organoids from day 8 to 18 (40- to 90-fold), whereas expression was not consistently observed in the monolayer cultures and day 3 organoids, highlighting the critical impact of the rotation culture system in Sertoli cell maturation. Furthermore, Leydig cell markers *HSD3B1*, *CYP17A1*, and *HSD17B3* were upregulated in day 3 organoids and exhibited stable expression in subsequent rotation culture systems (Figure 2I, Table S20). Immunofluorescence analyses further confirmed the presence of bipotential gonad markers GATA4 and WT1, as well as Sertoli cell marker SOX9, Leydig cell marker HSD3B1, and peritubular myoid cell marker α-SMA on days 3, 8, 13, and 18, indicating the stable maintenance of the organoid state throughout the rotation culture (Figure 2J-M, Figure S2F-G).

In summary, hiPSC-derived precursor testicular cells exhibited the capability to self-assemble into cell spheroids within a combined hanging drop and rotation culture system. The resulting testicular organoids maintained a stable state for up to 18 days concerning morphology, gene dynamics, and protein profiles.

### Characterization of hiPSC-derived testicular organoids

**Figure 3:**
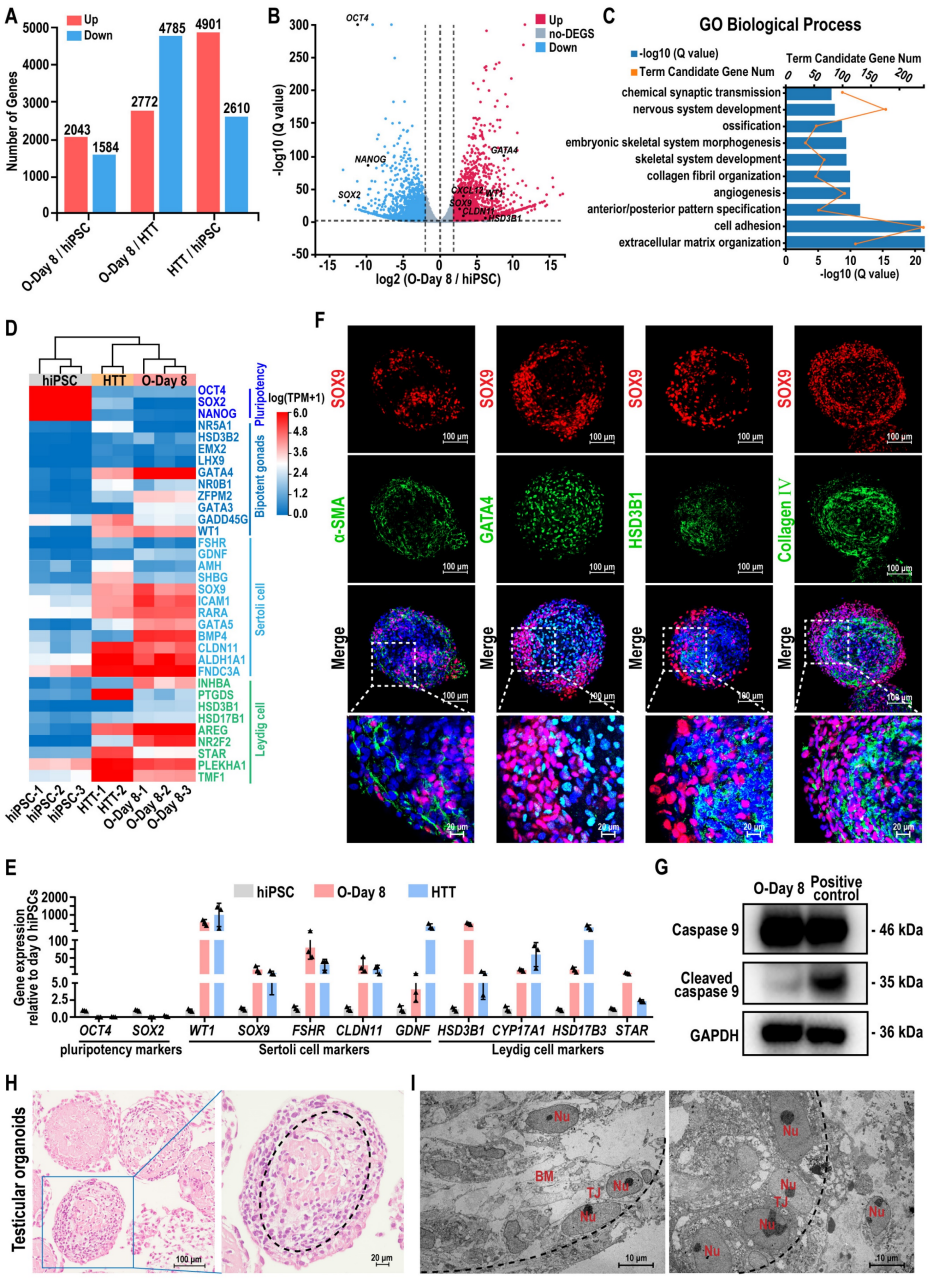
** Transcriptome analysis and characterization of hiPSC-derived testicular organoids. (A)** Number of up- or down-regulated genes (|log_2_FoldChange| ≥ 2, Q value ≤ 0.01) between hiPSC, day 8 testicular organoids (O-Day 8), and adult human testicular tissue (HTT). **(B)** Volcano plot of DEGs expression between O-Day 8 and hiPSC (|log_2_FoldChange| ≥ 2, Q value ≤ 0.01; up-regulation: red; down-regulation: blue). **(C)** GO term analyses of the top 10 GO categories in day 8 organoids compared with hiPSC. **(D)** Heatmap indicating the expression levels of marker genes for hiPSC, bipotent gonads, Sertoli cells, and Leydig cells. The relative gene expression level was indicated as red (up-regulated) or blue (down-regulated). **(E)** qRT-PCR data of relative gene expression in hiPSCs, O-Day 8 and HTT for pluripotency markers (*OCT4* and *SOX2*), Sertoli cell markers (*WT1*, *SOX9*,* FSHR*, *CLDN11*, and *GDNF*), and Leydig cell markers (*HSD3B1*,* CYP17A1*, *HSD17B3*, and *STAR*). The mRNA copy number of each gene was normalized with *GAPDH*. Gene expression was quantified relative to day 0 hiPSCs (mean ± SD, n = 3 independent experiments). **(F)** Coimmunostaining analysis was performed on day 8 organoids. Sertoli cells: SOX9. peritubular myoid cells: α-SMA. Bipotent gonad cells: GATA4. Leydig cells: HSD3B1. Basement membrane: Collagen IV. Nuclei were stained with DAPI (blue). The bottom images show a magnified view of the white dashed square in each merged figure. Scale bars = 100 µm, and 20 µm in magnified regions. **(G)** The apoptosis-related protein Caspase 9 in the testicular organoids cultured for 8 days was analyzed by Western blot assays. Testicular organoids treated with 50 µM Etoposide for 24 h were used as a positive control. **(H)** Paraffin sections of day 8 organoids stained with H&E for internal morphologic characterization. Scale bars, 100 μm and 20 μm. **(I)** The ultrastructure of day 8 organoids was detected by transmission electron microscopy. Nu, nucleus; TJ, tight junction; BM, basement membrane. Scale bars, 10 μm. The black dotted lines in (H) and (I) outline the cell arrangement pattern in the testicular organoid.

To analyze the gene expression profiles of hiPSCs and the hiPSC-derived testicular organoids, we employed RNA sequencing (RNA-seq). This method allowed for the comparison of gene expression profiles between hiPSCs and the testicular organoids (O-Day 8) with adult human testicular tissue (HTT), facilitating the investigation of global transcriptome changes during organoid formation. Among the 18,991 genes detected, 3627 genes exhibited more than a 2-fold differential expression between the testicular organoids and hiPSC, comprising 2043 genes that were up-regulated and 1584 genes that were down-regulated in the testicular organoids. When comparing the testicular organoids to HTT, 7557 genes exhibited more than a 2-fold differential expression, with 2772 genes up-regulated and 4785 genes down-regulated (Figure 3A). The volcano plot visualization indicated that most Sertoli cell and Leydig cell markers were activated in testicular organoids, whereas hiPSC markers were silenced (Figure 3B). Genes that were differentially expressed during the transition from hiPSC to testicular organoids were significantly enriched in Gene Ontology (GO) terms associated with testicular somatic cell functions, such as “extracellular matrix organization”, “cell adhesion” and “collagen fibril organization” (Figure 3C). Next, a testis-specific gene set of 656 genes was selected for unbiased hierarchical clustering, which was visualized using heatmaps. The unbiased hierarchical clustering revealed that although testicular organoids exhibited low expression of genes related to spermatogenesis, such as “spermatogenesis”, “spermatid development”, and “meiotic cell cycle”, they showed high expression of genes associated with male gonadal development and clustered together with HTT, while remaining distinct from hiPSCs. Additionally, genes highly expressed in both testicular organoids and HTT were enriched in GO terms, such as “positive regulation of male gonad development”, “negative regulation of female gonad development”, and “gonadal mesoderm development” (Figure S3A). Furthermore, the heatmap and qRT-PCR further revealed that the expression of key cell-specific markers for hiPSCs, bipotent gonad cells, Sertoli cells, and Leydig cells was highly concordant between testicular organoids and HTT, in contrast to hiPSC which showed significantly different gene expression (Figure 3D-E). These results suggested that hiPSC-derived testicular organoids contained testicular somatic-like cells, which could provide support for spermatogenesis.

Next, we performed IF staining to characterize their internal morphology (Figure 3F). The staining results for cross-sections of organoids revealed that SOX9-positive Sertoli cells and α-SMA-positive peritubular myoid cells were located at the periphery of the organoids, forming tubule-like characteristics similar to seminiferous tubules. GATA4-positive bipotent gonad cells and HSD3B1-positive Leydig cells were located in the interior compartment along the medial side of SOX9-positive Sertoli cells. Collagen IV-positive basement membrane was located on the inner side of the organoid surrounded by SOX9-positive Sertoli cells at the intercompartmental boundary, indicating Sertoli cell function 44. Quantitative analyses of immunofluorescence staining revealed that 44.71 ± 6.22% of the cells expressed SOX9, 26.70 ± 2.47% of the cells expressed GATA4, 23.76 ± 3.62% of the cells expressed α-SMA, 13.73 ± 2.58% of the cells expressed HSD3B1, and 37.96 ± 1.68% of the cells expressed Collagen Ⅳ (Figure S3B). In addition, the apoptotic marker cleaved Caspase 9 revealed no obvious apoptosis in the organoids (Figure 3G). Histological analysis further revealed that the organoid morphology remained intact and uniform during the rotational culture process, and the internal cells displayed a certain arrangement pattern and exhibited a tendency to form testicular cord-like structures. And the dotted lines in the figure roughly outline the trend of the cell arrangement (Figure 3H). Transmission electron microscopy (TEM) further confirmed the presence of the basement membrane and a single layer of polarized epithelial-like cells connected by the tight junction (TJ) within the testicular organoids (Figure 3I).

Taken together, these data suggested that the testicular organoids closely recapitulate the gene expression profile of human testis tissue and exhibit testicular cord-like characteristics. Moreover, the spatial arrangement of different types of testicular cells within the organoids closely corresponds to their locations in authentic testis tissue.

### Single-cell RNA-seq reveals the cellular components of testicular organoids

**Figure 4:**
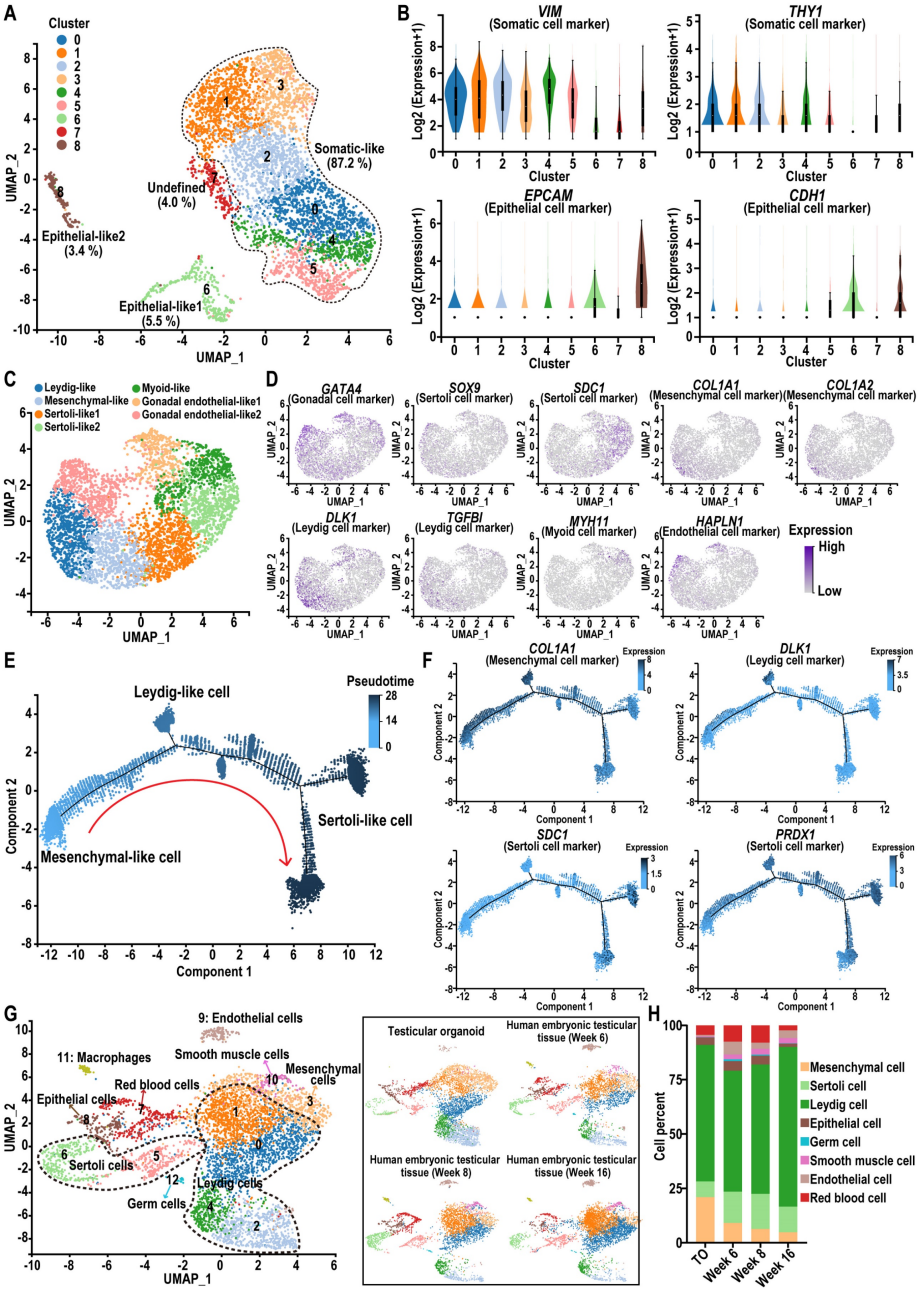
** Single-cell RNA-seq reveals cell-type heterogeneity in hiPSC-derived testicular organoids. (A)** UMAP plots of hiPSC-derived testicular organoids. The data represent 10,053 cells. Dozens of organoids were pooled for one scRNA-seq experiment. The cells were annotated into nine cell clusters. Dashed lines outline the cell populations unique to somatic-like cells. **(B)** The expression of somatic (*VIM* and *THY1*) and epithelial cell markers (*EPCAM* and *CDH1*) for the classification of cells in whole testicular organoid. **(C)** UMAP plots of somatic-like cells in testicular organoids. Data represent 8765 somatic-like cells. These cells were separated into seven clusters and annotated into five cell types. **(D)** Feature plots for the expression of specific marker genes in different cell types. For each cell type, 1-2 cell markers are shown in the main figure, accompanied by a gallery of additional markers in Figure S4A. The shades of purple in the UMAP plot reflect the relative expression levels of the corresponding genes. **(E)** Pseudotime trajectory (Monocle analysis) of somatic-like cells. Cells are colored according to their predicted position along pseudotime. **(F)** Feature plots for the expression levels of mesenchymal, Leydig, and Sertoli cell marker genes along the pseudotime axis. The color scale represents expression levels. **(G)** Integrative analysis of testicular organoid data with published scRNA-seq data from human embryonic testicular tissue at 6, 8, and 16 weeks postfertilization (GSE143356). Single-cell transcriptome data were subjected to dimensionality reduction via UMAP, where each dot represents a single cell (n = 23,808). The left panel is colored according to their cell type identity and labeled with the corresponding cell categories, while the right panel shows the cell type-specific distribution of each sample. For each cell type, 1-2 cell markers are shown in a marker gallery in Figure S4D. **(H)** The stacking plot shows the proportion of cell types in each sample.

To gain insight into the transcriptome heterogeneity and cellular composition of hiPSC-derived testicular organoids, we performed scRNA-seq of testicular organoids using the 10X Genomics platform. Unbiased clustering identified nine cell clusters in testicular organoids (Figure 4A). Clusters 6 and 8 contained *EPCAM* ^+^ and *CDH1* ^+^ cells that did not express *VIM* or *THY1*, indicating the presence of a small population of epithelial-like cells in the organoids (Figure 4B). The group of testicular somatic-like cells expressing *VIM* and *THY1* (initial clustering of clusters 0-5) was subsequently re-clustered into seven clusters, identified based on the expression of *GATA4* (Gonadal), *SOX9*, *SDC1* and *PRDX1* (Sertoli), *COL1A1* and *COL1A2* (Mesenchymal), *DLK1*, *TGFBI* and *INHBA* (Leydig), *MYH11* (Myoid), *HAPLN1* and *SLIT2* (Endothelial). A previous scRNA-seq study of human testis revealed that somatic niche cells in the adult testis include Sertoli cells, Leydig cells, myoid cells, endothelial cells, and macrophages, which provide physical and hormonal support for spermatogenesis 45. Consistent with these findings, the somatic-like cell group in testicular organoids was annotated into five major cell groups: Leydig-, mesenchymal-, Sertoli-, myoid-, and gonadal endothelial-like cells (Figure 4C-D, Figure S4A). In addition, no cell cluster expressed pluripotency markers such as *SOX2* and *OCT4* (Figure S4B-C). Additionally, we conducted pseudotime analysis to investigate whether organoids recapitulate the process of testicular organogenesis. Pseudotime analysis revealed one cell cluster at the early pseudotime that transcriptionally bifurcates into two different lineages, Leydig and Sertoli, in the late pseudotime (Figure 4E). Moreover, trajectory analysis revealed that the expression level of Sertoli cell marker genes increased along the pseudotime axis (Figure 4F). These results demonstrated the occurrence of cell differentiation in testicular organoids and are consistent with previous single-cell analyses of human testicular development, which indicated that Sertoli and Leydig cells may derive from a common heterogeneous progenitor pool 31.

Next, to confirm the developmental stage of the testicular organoids, we conducted an integrated analysis of the testicular organoid scRNA-seq data with a previously published dataset from human embryonic and fetal testicular tissue at 6, 8, and 16 weeks postfertilization (GSE143356) 31. The results showed that testicular organoid-derived cells closely clustered with primary human testis cells, with mesenchymal-, Leydig-, Sertoli-, endothelial-, and epithelial-like cells blended into their corresponding cell types in human testicular tissue in the UMAP plane, indicating that the gene expression profiles of these somatic cell types in testicular organoids were similar to those in primary testicular tissue (Figure 4G, Figure S4D). Specifically, we calculated the proportion of different cell types in each group. The results showed that the percentages of Leydig cells, Sertoli cells, and epithelial cells in the testicular organoids group were 62.80%, 7.19%, and 3.46%, respectively. In the week 8 group, these percentages were 59.41%, 16.13%, and 4.10%, respectively (Figure 4H). These findings indicated that the testicular organoids exhibited a similar proportion of certain cell types to those observed in the week 8 embryonic testicular tissue.

These findings suggested that the testicular organoids we constructed could effectively mimic the testicular niche at the 8-week postfertilization stage, exhibiting the potential to simulate the early testicular structure and the testicular somatic cell microenvironment.

### hiPSC-derived testicular organoids exhibit hormone responsiveness and blood-testis barrier function

**Figure 5:**
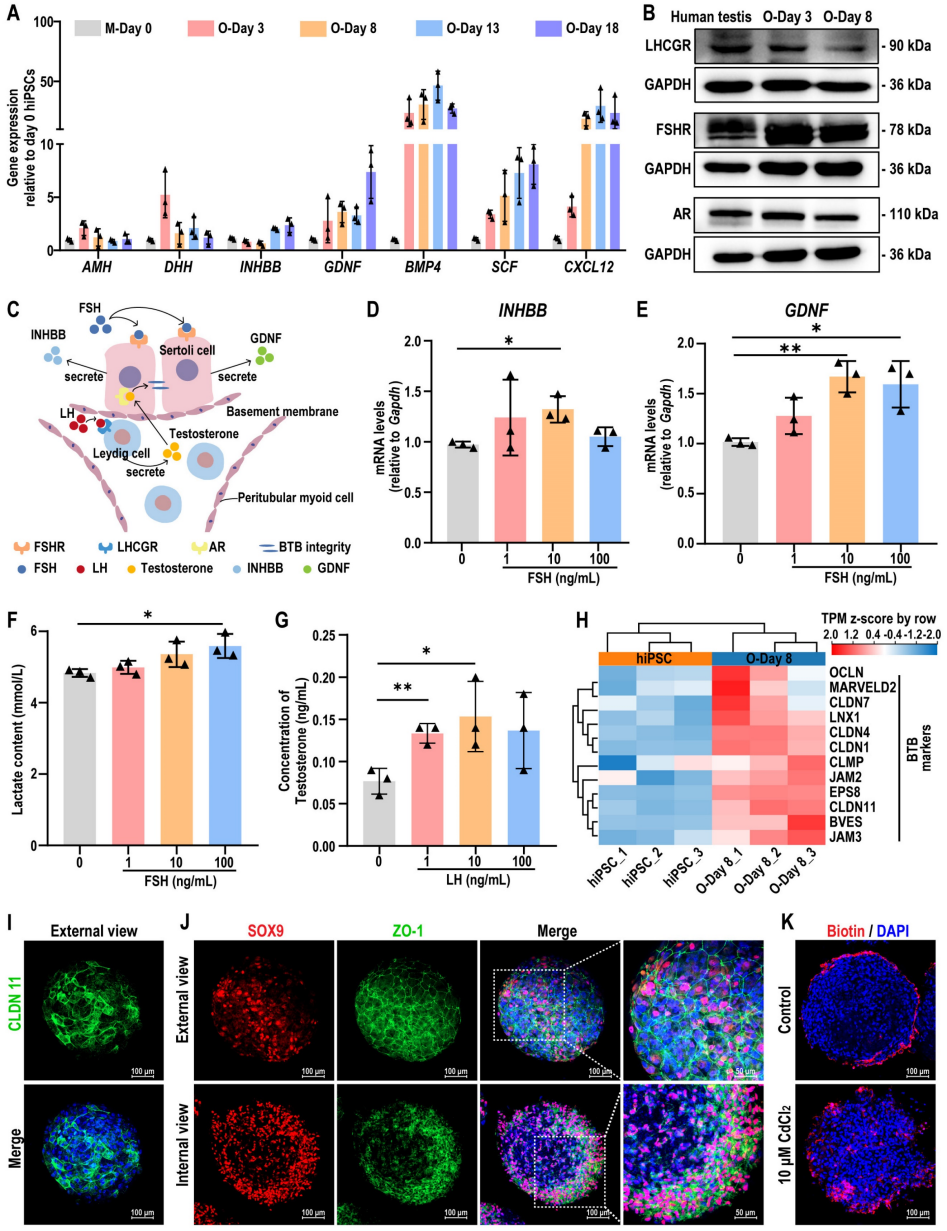
** Functional evaluation of testicular organoids. (A)** qRT-PCR data was collected to analyze the relative gene expression of cytokines in day 0 hiPSCs, as well as in organoids at days 3, 8, 13, and 18. The mRNA copy number of each gene was normalized with *GAPDH*, and gene expression levels were quantified relative to day 0 hiPSCs (mean ± SD, n = 3 independent experiments). **(B)** Protein expression of LHCGR, FSHR, and AR was analyzed by Western blot in organoids at days 3 and 8, and adult testicular tissue. **(C)** Schematic diagram of the interaction between sex hormones and receptors in the seminiferous tubules. **(D-E)** The expression levels of *INHBB* and *GDNF* in day 8 testicular organoids treated with FSH at concentrations of 0, 1, 10, and 100 ng/mL for 24 h were analyzed using qPCR. The mRNA copy numbers of each gene were normalized with *GAPDH*. **(F)** The lactate test kit was used to measure lactate concentration in the supernatants of day 8 testicular organoids induced with FSH at concentrations of 0, 1, 10, and 100 ng/mL. **(G)** Radioimmunoprecipitation assay (RIA) was employed to measure testosterone concentration in the supernatants of day 8 testicular organoids induced with LH (0, 1, 10, and 100 ng/mL). The data were from three independent experiments and were expressed as mean ± SD, *P < 0.05; **P < 0.01. **(H)** Heatmap image of a set of BTB tight junction-related genes in hiPSC and day 8 organoids. Red and blue indicate up-and down-regulated genes, respectively. **(I)** The expression of CLDN11 in day 8 organoids was examined by immunofluorescence. **(J)** Coimmunostaining analysis was used to examine the colocalization of SOX9 and ZO-1 in day 8 organoids. The upper panel displays the external view of the organoid (top-down view), while the lower panel shows the internal view of the organoid (cross-section). The rightmost images show an enlargement of the white dashed square in the merged figure. Scale bars = 100 µm, and 50 µm in magnified regions. **(K)** After treating day 8 organoids with 10 μΜ CdCl_2_ for 24 h, the BTB integrity of the organoids was evaluated using the sulfo-NHS-LC-biotin assay. Scale bars, 100 μm.

Sertoli cells secrete chemokines and cytokines that are essential for the maintenance and differentiation of SSCs in the testis. Therefore, we analyzed the expression of cytokines such as *AMH*, *DHH*, *INHBB*, *GDNF*, *BMP4*, *SCF*, and *CXCL12* using qRT-PCR at various time points during organoid culture. The results showed that these cytokines were induced in organoids, with their expression levels higher than those in day 0 hiPSCs. Expression levels increased with extended induction time and stabilized between day 8 (O-Day 8) and day 18 (O-Day 18) of rotational culture (Figure 5A).

Notably, the downregulation of *AMH* (a marker for immature Sertoli cells) in organoids, suggested the gradual maturation of Sertoli cells within the rotational culture system of organoids. To verify whether testicular organoids exhibit responsive characteristics to hypothalamic-pituitary-gonadal axis (HPG axis) regulation under *in vitro* culture conditions, we analyzed the expression of LHCGR, FSHR, and AR on day 3 (O-Day 3) and day 8 (O-Day 8) of testicular organoids, and compared them with adult human testicular tissue. The results showed that these sex hormone receptors were expressed in organoids, with expression patterns consistent with those observed in testicular tissue (Figure 5B, Figure S10). FSH and LH are gonadotropins produced by the pituitary gland in response to GnRH. LH binds to LHCGR on Leydig cells, inducing testosterone secretion, which then binds to AR in Sertoli cells to maintain the integrity of the BTB. FSH binds to FSHR on Sertoli cells, stimulating the secretion of INHBB and GDNF (Figure 5C). Additionally, FSH also prompts Sertoli cells to secrete lactate, the primary energy source for germ cells. Normal testicular function depends on the coordinated action of all these hormones.

To evaluate whether testicular organoids exhibit hormone responsiveness similar to that of testes, we examined the expression of *INHBB* and *GDNF*, as well as lactate production, following treatment with different concentrations of FSH. The results indicated that FSH significantly increased the expression levels of *INHBB* and *GDNF*, and enhanced lactate production (Figure 5D-F). Furthermore, LH treatment promoted testosterone secretion in the organoids, indicating that these organoids exhibited characteristics similar to Leydig cells (Figure 5G). In summary, these findings demonstrated that testicular organoids were regulated by gonadotropins and exhibited endocrine functions.

Next, we investigated whether testicular organoids formed a functional BTB. To assess this, we examined the expression changes of genes specific to tight junctions of BTB within the organoids. Transcriptome sequencing revealed that the genes involved in tight junction formation were activated in day 8 organoids (Figure 5H). Additionally, immunofluorescence staining was used to detect the expression and localization of CLDN11 and ZO-1, a scaffolding protein in tight junctions. The results showed that SOX9-positive Sertoli cells were arranged in a continuous layer surrounding the organoids, and CLDN11 and ZO-1 were neatly localized at cell-cell interfaces, supporting the integrity of the tight junction barrier (Figure 5I-J). To further evaluate BTB functionality, we treated the organoids with sulfo-NHS-LC-biotin, a water-soluble and membrane-impermeant biotinylation reagent, to analyze BTB permeability. We found that sulfo-NHS-LC-biotin could not penetrate the interior of the organoids, suggesting that the organoids formed an intact BTB, mimicking the *in vivo* BTB. When the organoids were treated with CdCl_2_, a compound known to induce reproductive toxicity, sulfo-NHS-LC-biotin was observed within the organoids, indicating a disruption of the BTB tight junctions (Figure 5K). This finding suggested that the BTB in organoids had the potential to model drug-induced damage to the BTB.

In summary, hiPSC-derived testicular organoids exhibited endocrine functions, maintained gonadotropin responsiveness, and formed functional BTB. These features render them valuable tools for assessing the impacts of various drugs and compounds on testicular function.

### Assessment of testicular organoids as a model for studying the relationship between semaglutide and testicular function

The observation of the BTB and interactions between testicular somatic cells in our testicular organoids provides a model for studying drug effects within the testis. To validate the reliability of testicular organoids as a drug screening and evaluation model, we exposed the testicular organoids to three known reproductive toxicity: CdCl_2_, etoposide, and doxorubicin. The results showed that increasing the concentration of these compounds led to detrimental effects on organoid morphology, cell viability, and BTB function, demonstrating the organoids' sensitivity to toxic agents and their potential use in evaluating drug-induced testicular dysfunction (Figure S6).

**Figure 6:**
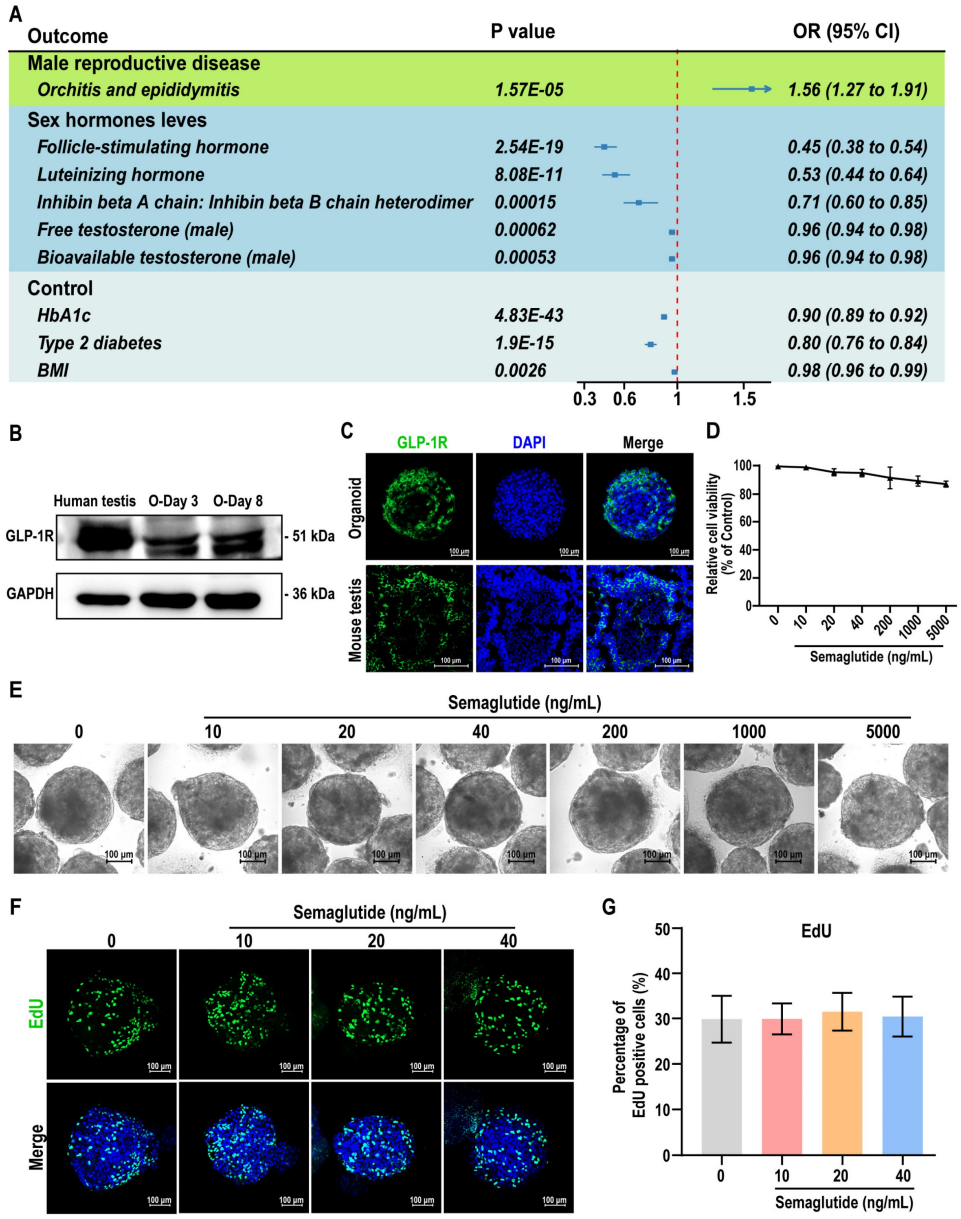
** Testicular organoids served as a model for the evaluation of semaglutide, a GLP-1R agonist. (A)** Results of two-sample MR analysis of GLP-1R and male reproductive disease risk and sex hormone levels. **(B)** Protein expression of GLP-1R in adult human testicular tissue and days 3 and 8 organoids was assessed using Western blot analysis. GAPDH was utilized as the loading control. **(C)** The expression of GLP-1R in testicular organoids at day 8 and mouse testes at week 8 was analyzed by immunofluorescence. Nuclei were counterstained with DAPI (blue). Scale bar, 100 µm. **(D)** The cell viability of testicular organoids treated with different concentrations of semaglutide (0, 10, 20, 40, 200, 1000, and 5000 ng/mL) for 24 h was measured by CCK8. **(E)** Representative bright-field images showed organoids after 24 h of exposure to different concentrations of semaglutide. Scale bar, 100 µm. **(F)** EdU staining (green) detected the proliferation of organoids following a 24 h treatment with semaglutide at 0, 10, 20, and 40 ng/mL. DAPI as a nuclear stain (blue). Scale bar, 100 µm. **(G)** Semi-quantitative analysis of EdU-positive cells in (F) was performed using ImageJ software. The data are expressed as mean ± SD.

Given the controversial role of semaglutide, a GLP-1 receptor agonist (GLP-1 RA), on testicular function, we further investigated its effects on testicular organoids. First, we performed a drug target Mendelian Randomization (MR) analysis to explore potential associations between GLP-1 RA exposure and male reproductive diseases or sex hormone levels. A total of 22 significant cis-eQTLs from eQTLGen were selected as genetic instrumental variables (IVs) for the GLP-1 receptor gene (GLP-1R), following the methodology of Sun *et al.* 32. The average F-statistic for these IVs was 51, indicating no bias due to weak instruments (see Table S3). Notably, genetically-proxied GLP-1 RA exposure was significantly associated with decreased HbA1c levels [OR (95%) = 0.90 (0.89-0.92), P < 0.001], reduced type 2 diabetes risk [OR (95%) = 0.80 (0.76-0.84), P < 0.001], and lower BMI [OR (95%) = 0.98 (0.96-0.99), P = 0.0026], confirming the validity of the IVs (see Table S5). However, inverse variance weighted (IVW)-MR analysis revealed that GLP-1 RA was associated with an increased risk of orchitis and epididymitis [OR (95%) = 1.56 (1.27-1.91), P < 0.001] (Figure 6A). Additionally, increased GLP-1R gene expression correlated with decreased levels of FSH [OR (95%) = 0.45 (0.38-0.54), P < 0.001], LH [OR (95%) = 0.53 (0.44-0.64), P < 0.001], INHBB [OR (95%) = 0.71 (0.60-0.85), P < 0.001], free testosterone [OR (95%) = 0.96 (0.94-0.98), P < 0.001], and bioavailable testosterone [OR (95%) = 0.96 (0.94-0.98), P < 0.001] (Figure 6A, also see Table S6). The consistency between the IVW and median weighting methods (see Table S7), combined with the absence of heterogeneity (Cochrane Q test) and horizontal pleiotropy (MR-Egger intercept test) (see Table S8 and Table S9), confirmed the association between GLP-1 RA exposure and altered testicular hormone secretion.

To experimentally validate the effects of GLP-1 RA exposure on testicular hormone function, we treated the testicular organoids with semaglutide. Western blot and immunofluorescence analyses confirmed the expression of GLP-1R in the organoids, with control mouse testis tissue showing co-expression of GLP-1R in Sertoli and Leydig cells (Figure 6B-C, Figure S11). We then evaluated the effects of semaglutide on organoid viability and proliferation using CCK8 and EdU assays. Semaglutide, at concentrations below 40 ng/mL, did not significantly affect the morphology, viability, or proliferation of the organoids (Figure 6D-G). These results demonstrated that testicular organoids expressed GLP-1R, the target of GLP-1 RAs, and that semaglutide does not exhibit notable toxic effects on organoid viability or proliferation. This result ruled out the possibility that semaglutide affected testicular hormone secretion through the suppression of cell viability or proliferation.

### Semaglutide inhibits testosterone production without compromising BTB integrity in testicular organoids

**Figure 7:**
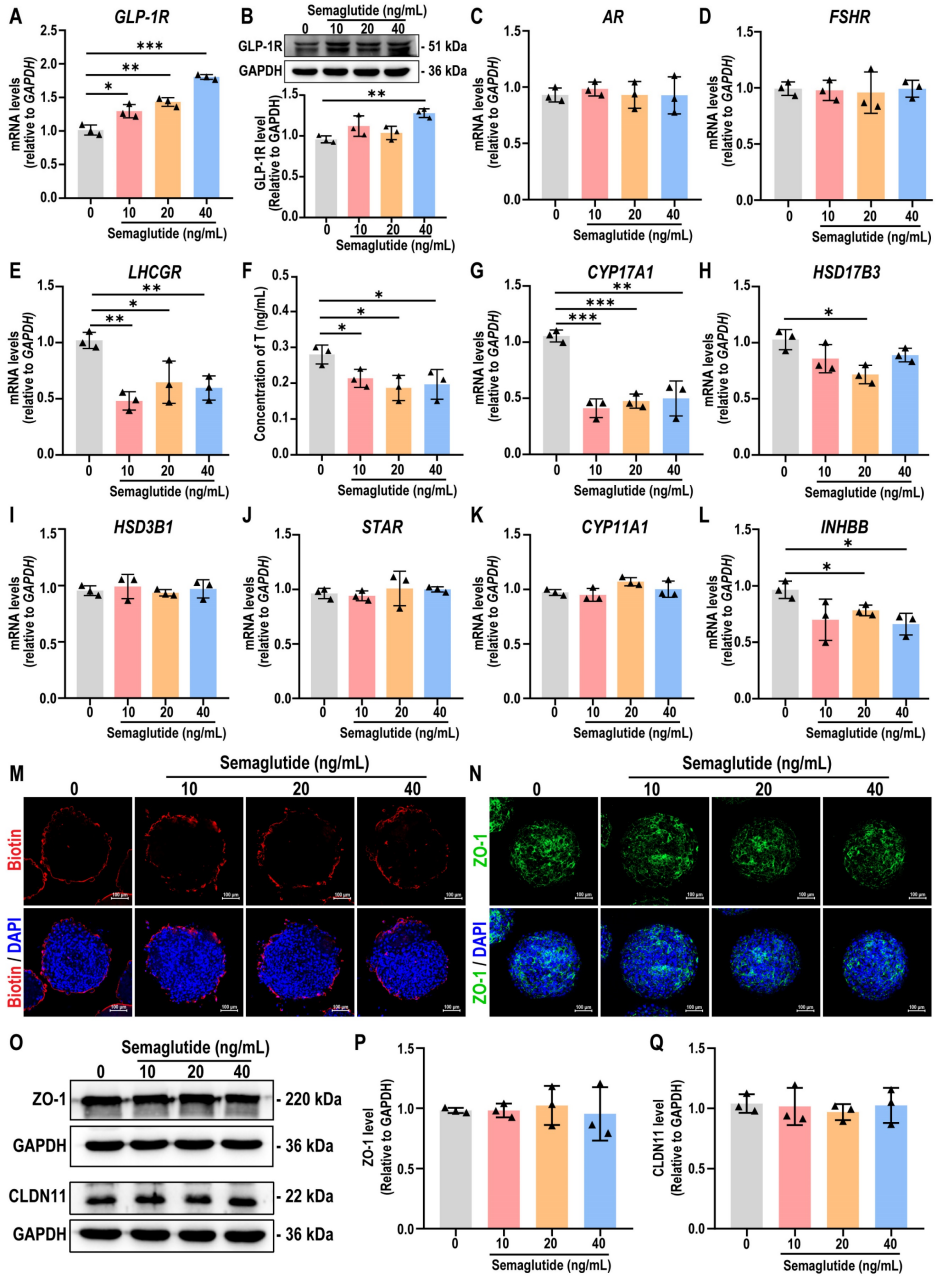
** Semaglutide significantly reduced testosterone secretion in testicular organoids. (A)** The expression level of *GLP-1R* in organoids treated with semaglutide (0, 10, 20, and 40 ng/mL) for 24 h was detected by qPCR. The mRNA copy number of each gene was normalized with *GAPDH*. **(B)** The protein expression of GLP-1R in day 8 organoids treated with semaglutide for 24 h was analyzed using Western blot. GAPDH was utilized as the loading control. **(C-E)** The expression levels of *AR*, *FSHR*, and* LHCGR* in day 8 organoids treated with semaglutide for 24 h were assessed using qPCR. The mRNA copy numbers for each gene were normalized against *GAPDH*. **(F)** RIA assay was employed to measure testosterone concentration in the supernatants of testicular organoids treated with semaglutide at concentrations of 0, 10, 20, and 40 ng/mL. **(G-K)** The expression levels of key genes involved in the testicular steroidogenesis pathway, including *CYP17A1*, *HSD17B3*, *HSD3B1*, *STAR*, and *CYP11A1*, were analyzed in organoids treated with semaglutide for a duration of 24 h using qPCR. **(L)** The expression levels of *INHBB* in organoids treated with semaglutide for 24 h were analyzed using qPCR. The mRNA copy numbers of each gene were normalized with *GAPDH*. **(M)** After treating day 8 organoids with 0, 10, 20, and 40 ng/mL semaglutide for 24 h, the integrity of BTB in the organoids was assessed using the sulfo-NHS-LC-biotin assay. **(N)** Immunofluorescence was employed to detect ZO-1 protein expression in day 8 organoids exposed to semaglutide for 24 h. Nuclei were counterstained with DAPI (blue). Scale bar, 100 µm. **(O-Q)** Protein expression of ZO-1 and CLDN11 was analyzed by Western blot in day 8 organoids treated with semaglutide for 24 h. GAPDH served as the loading control. All data were from three independent experiments and were expressed as mean ± SD, *P < 0.05; **P < 0.01; ***P < 0.001.

Next, we evaluated the expression of *GLP-1R* and hormone-related receptors *AR*, *FSHR*, and *LHCGR* in testicular organoids following semaglutide treatment. The results demonstrated that semaglutide enhanced the expression of *GLP-1R*, as well as the known *GLP-1R* downstream markers *KISS1* and *KISS1R* genes, in organoids in a dose-dependent manner, confirming that the organoids were responsive to semaglutide (Figure 7A, Figure S7A-B). Western blot analyses also corroborated this finding (Figure 7B). Furthermore, semaglutide treatment significantly reduced the expression of *LHCGR*, a crucial receptor for testosterone synthesis, while the expression of *AR* and *FSHR* remained unaffected (Figure 7C-E). To assess the impact of semaglutide on Leydig cell function, testosterone levels were measured in the organoids. The findings confirmed that semaglutide significantly inhibited testosterone synthesis, consistent with the trend observed in the MR analysis results (Figure 7F). qPCR analysis further revealed that semaglutide significantly downregulated the expression levels of *CYP17A1* and *HSD17B3*, key genes involved in testicular steroidogenesis, whereas the expression of *HSD3B1*, *STAR*, and *CYP11A1* remained unchanged (Figure 7G-K).

Given that testosterone directly influences Sertoli cell function by binding to AR, we next examined the expression changes of *INHBB* in testicular organoids following semaglutide treatment. The results indicated that semaglutide significantly downregulated the expression level of *INHBB* (Figure 7L). Additionally, since the testicular organoids possess functional BTB structures, we assessed the impact of semaglutide on BTB integrity. The sulfo-NHS-LC-biotin assay experiments, as well as immunofluorescence and western blot analyses revealed that semaglutide did not disrupt the BTB permeability, nor did it downregulate the expression of BTB key protein ZO-1 and CLDN11, indicating that semaglutide did not affect the BTB function of testicular organoids (Figure 7M-Q, Figure S12).

These findings suggested that semaglutide modulated gene expression related to hormone synthesis, leading to inhibited testosterone production and influencing Sertoli cell function, while preserving the structural and functional integrity of the BTB in testicular organoids.

### Identify key targets of reprogramming compounds and investigate potential mechanisms for inducing testicular organoids from hiPSCs

**Figure 8:**
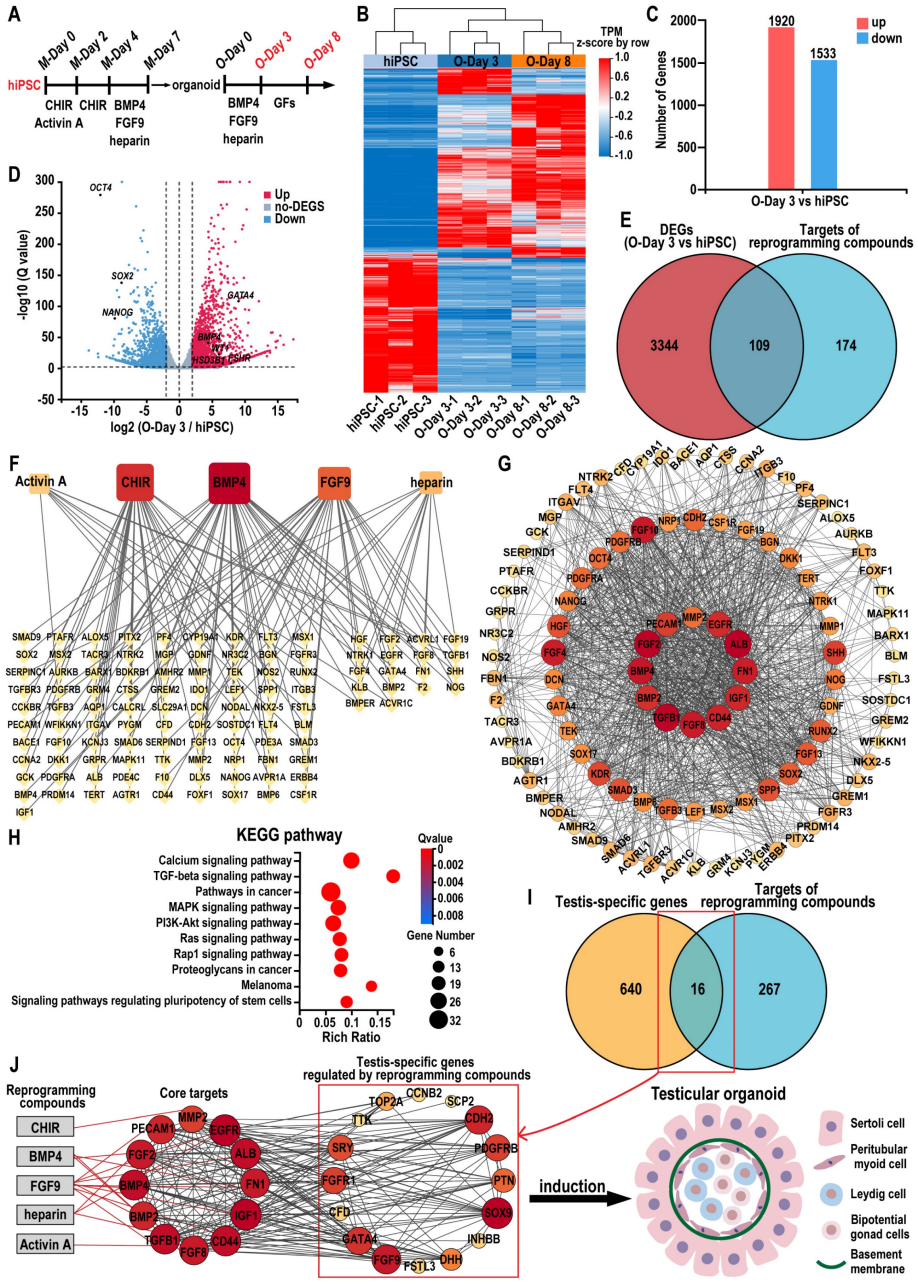
** Combining network pharmacology and bioinformatics to identify reprogramming mechanisms during testicular organoid induction. (A)** Schematic diagram of the initial hiPSCs-directed differentiation protocol for inducing testicular organoids. GFs: growth factors. **(B)** Heatmap of microarray data of hiPSC, day 3 organoids and day 8 organoids (|log_2_FoldChange| ≥ 2, Q value ≤ 0.01). Blue indicates decreased expression, and red indicates increased expression. **(C)** Number of up- or down-regulated genes between day 3 testicular organoids and hiPSC (|log_2_FoldChange| ≥ 2, Q value ≤ 0.01). **(D)** Volcano plot of the DEGs between day 3 organoids and hiPSC (|log_2_FoldChange| ≥ 2, Q value ≤ 0.01; up-regulation: red; down-regulation: blue). **(E)** The Venn diagram analysis of the DEGs (day 3 organoids vs hiPSC) and the predicted targets of reprogramming compounds (CHIR, Activin A, BMP4, FGF9 and heparin). **(F)** The network of the relationship between the reprogramming compounds and 109 common targets. Yellow diamond nodes represent targets, and square nodes represent reprogramming compounds. Lines represent interactions between compounds and targets; the size and color of reprogramming compound nodes are proportional to the number of associated targets. **(G)** The PPI network of common targets for the induction of testicular organoids. The circles represent proteins, the colors (from yellow to orange to red) indicate the degree of binding between the proteins. The lines represent protein-protein interactions. **(H)** The KEGG enrichment analyses of 109 common targets (the top ten results). **(I)** The Venn diagram analysis of 656 testis-specific genes and 283 predicted targets of reprogramming compounds. **(J)** Reprogramming compound-core gene-testis network diagram. Gray rectangles represent compounds, and circles represent genes. Red lines represent interactions between compounds and targets, and black lines represent interactions between genes.

To elucidate the role of the combination of five reprogramming compounds—CHIR, activin A, BMP4, FGF9, and heparin—in specifying testicular organoids, we analyzed gene expression profiles in hiPSC, day 3 organoids (O-Day 3), and day 8 organoids (O-Day 8). Hierarchical clustering analysis revealed that the transcriptome profiles of O-Day 3 and O-Day 8 were distinct from those of hiPSC, indicating that the combination of reprogramming compounds induced global transcriptional changes in hiPSCs towards a testicular cell fate (Figure 8A-B). Among the 18,991 genes analyzed, 3453 genes were differentially expressed (≥ 2-fold change) between hiPSC and O-Day 3, with 1920 upregulated and 1533 downregulated genes in O-Day 3 (Figure 8C). The volcano plot demonstrated that the reprogramming compounds combination downregulated pluripotency genes in O-Day 3, such as *OCT4*, *SOX2*, and *NANOG*, while activating gonadal cell-specific genes, including *GATA4*, *WT1*, *BMP4*, *FSHR*, and *HSD3B1* (Figure 8D). GO analysis indicated that the upregulated genes during the transition from hiPSC to O-Day 3 were enriched in biological processes such as extracellular matrix organization and cell adhesion, which are related to testicular somatic cell function (Figure S8A).

The DEGs between O-Day 3 and O-Day 8 were significantly less than those between O-Day 3 and hiPSC, suggesting that the combination of reprogramming compounds played a crucial role in the transition from hiPSC to O-Day 3 (Figure S8B). We then predicted the molecular targets of the five reprogramming compounds using SwissTargetPrediction, TTD, DrugBank, ChEMBL, STITCH, and STRING databases (see Tables S10-S14). After eliminating duplicates, 283 potential targets were identified (see Table S15). Venn analysis revealed 109 common targets between the compound-related targets and the 3453 DEGs from hiPSCs to O-Day 3 transition (Figure 8E). A regulatory network of the reprogramming compound combinations and the common targets was constructed using Cytoscape software. In the network, the degree value represents the number of edges connected to each node, with the size and color of the node being proportional to its degree value. We found that the top three compounds in terms of degree were BMP4, CHIR, and FGF9, suggesting their pivotal role in testicular organoid specification (Figure 8F). Further analysis of the 109 common targets through STRING's PPI network, visualized via Cytoscape. The screening conditions were set to degree > 35 and betweenness centrality > 0.024, resulting in 12 core target genes ranked from largest to smallest by node degree: ALB, TGFB1, FGF2, BMP4, FGF8, FN1, CD44, IGF1, EGFR, BMP2, PECAM1, and MMP2 (Figure 8G, also see Table S16). To explore the underlying mechanism, we performed KEGG and GO enrichment analysis on 109 common targets. KEGG enrichment analysis revealed that these targets were predominantly associated with calcium signaling pathway, TGF-β signaling pathway, MAPK signaling pathway, and signaling pathways regulating pluripotency of stem cells (Figure 8H). In terms of biological processes, the targets were enriched in BMP signaling pathway, positive regulation of MAPK cascade, and cell migration, etc. (Figure S8C). In a similar vein, by intersecting 7511 DEGs in human testis samples (HTT vs hiPSC) with reprogramming compound-related targets, we identified 125 overlapping targets in the human testis (Figure S8D). Through the same type of network analysis as conducted for organoids, we obtained 9 core target genes, four of which—ALB, FGF2, IGF1, and FN1—were also core targets in the reprogramming compound-induced organoid generation (Figure S8E). Furthermore, the overlap rate of the KEGG enrichment analysis results for these 125 intersection genes from human testis and the 109 genes that induced organoids reached 80% (Figure S8F). These findings suggested that the pathways regulated by reprogramming compounds in testicular organoid induction are analogous to those in the human testis.

Additionally, to further elucidate how reprogramming compounds induce hiPSCs into testes, the 283 reprogramming compounds-related targets with a testis-specific gene set (656 genes) identified 16 testis-specific genes regulated by these compounds (Figure 8I). The compounds-core targets-testis network constructed in Cytoscape depicted the overall process of reprogramming compound-driven testicular organoid induction (Figure 8J). This constructed network highlights 12 core target genes such as ALB, TGFB1, FGF2, BMP4, FGF8, FN1, CD44, IGF1, EGFR, BMP2, PECAM1, and MMP2, as pivotal in the differentiation process. These critical roles were further supported by KEGG and GO enrichment analyses, which identified pathways essential for cell differentiation and organogenesis, specifically highlighting the BMP and MAPK signaling pathways. The culmination of these pathways' interactions ultimately leads to the formation of a structured testicular organoid comprising Sertoli cells, peritubular myoid cells, Leydig cells, bipotential gonad cells, and a basement membrane.

## Discussion

In this study, we proposed a feeder-free protocol to direct human iPSCs toward the formation of complex multicellular testicular organoids. This was achieved by carefully balancing the anterior and posterior modes of the intermediate mesoderm (IM) with small molecules, and employing a previously established culture system that integrates hanging drops and rotation. The organoids displayed a cell aggregation pattern with certain testicular cord-like structures and successfully recapitulated key *in vivo* testicular functions, such as the tight junction function of blood-testis barrier (BTB) and hormone responsiveness. In contrast to previous methods for inducing testicular organoids from hESCs 46, the organoids developed in this study circumvent ethical and legal issues.

*Ex vivo* testicular organogenesis from hPSCs presents significant challenges due to the intricate structure and function of testes. Previous research by Pryzhkova and Jordan demonstrated a significant advancement in this area by utilizing a mini-spin bioreactor to co-culture hESCs-derived spheroids and adult testicular somatic cells, resulting in the generation of testicular organoids and reconstruct the adult testicular niche 47. However, the limited availability of testicular donor tissue and the ethical restrictions surrounding hESCs pose challenges to the scale-up of organoid production, potentially hindering further functional assessments and downstream translational applications 48, 49. To circumvent ethical and donor limitations, we employed the previously developed hanging drop in conjunction with rotation culture system. This approach enabled us to derive human testicular organoids relying on the differentiation potential of hiPSCs and their self-organization capabilities within a 3D culture environment. Compared to earlier studies, our method is easy to use, rapid, and reproducible 50. For instance, while the testicular organoids generated by Oliver *et al.* exhibited similar morphology, their method involved a complex three-layer gradient system and required labor-intensive growth in additional hanging cell inserts 50. In contrast, our protocol formed organoids in hanging drops, allowing for precise size control and effortless transfer to Erlenmeyer flasks with minimal effort. Furthermore, we observed the rapid formation of testicular cord-like structures, which were visualized through immunofluorescence as early as day 8 of organoid culture, which significantly faster than other methods that require at least 16 days 29, 46. Cell density is also a critical factor in the preparation of testicular organoids. Oliver *et al.* demonstrated that larger and more complex organoid structures can be formed in a three-layer gradient system with 132,000 human primary testicular cells per drop, which likely enhanced cell-cell interactions and paracrine communication 50. However, the limited availability of cells from human samples presents challenges in scaling up testicular organoids of high cell densities. In our study, testicular organoids were prepared at a relatively low cell density (5000 cells/drop), which improved nutrient and oxygen perfusion within the organoids. In contrast to a three-layer gradient system suspended in a 24-well plate, our rotational culture system continuously supplies fresh medium to the organoids and increases the contact area available for nutrient and oxygen diffusion 51. And no significant decrease in organoid viability was observed even on day 33 of culture. This streamlined approach not only simplifies the organoid generation process but also enhances the efficiency and reproducibility of testicular organoid formation.

It is well known that the bipotent gonads develop from the genital ridge inside the intermediate mesoderm (IM) and then differentiate into testes determined by the presence or absence of the Y-chromosome testis-determining gene SRY 52. Consistent with the intrinsic developmental pathway, the development of hiPSCs into specific lineages or organs involves transitioning through various developmental stages, the first of which is differentiation into three germ layers 53, 54. Directed differentiation of hiPSCs into IM and testicular lineages can be achieved by overexpressing reprogramming factors or by adding specific reprogramming compounds or growth factors to the culture medium at precise time points 28, 29, 55. This *in vitro* directed differentiation is efficient and controllable, avoiding possible interference during *in vivo* development and not requiring coordination of the overall development of the individual, thus having significant advantages in areas such as drug screening and personalized medicine. The selection of the reprogramming compounds is crucial for successful differentiation. In our study, we identified a well-characterized group of compounds, including CHIR-99021, activin A, BMP4, FGF9, and heparin, which can induce pluripotent stem cell differentiation and effectively regulate various signaling pathways involved in this process 56. CHIR-99021 is an inhibitor of glycogen synthase kinase 3 (GSK3) that activates the canonical Wnt signaling pathway and is commonly employed to drive the induction of mesendoderm specification 57. Activin A, a member of the TGF-beta superfamily, plays a role in a wide array of biological processes such as stem cell proliferation, differentiation, cell fate determination, and organogenesis 58. BMP4 is known to induce mesodermal signals that are essential for the development of the urogenital system and enhance the expression of SOX9 59, 60. FGF9, expressed in Sertoli cell precursors, can promote sustained expression of SOX9 via fibroblast growth factor receptor (FGFR) as a paracrine factor 61, 62. Additionally, heparin is necessary for the efficient activation of FGFR by FGF and serves as an accessory molecule to modulate FGF binding and FGFR activation 63-65. The sustained increase of SOX9 expression observed in our testicular organoids was consistent with the effective stimulation of all reprogramming compounds administered. The PPI network was constructed based on the 109 potential targets, leading to the identification of 12 core targets (ALB, TGFB1, BMP4, BMP2, FGF2, FGF8, FN1, CD44, IGF1, EGFR, PECAM1, and MMP2). KEGG enrichment analysis of the 109 potential targets indicated that the molecular mechanisms underlying the formation of testicular organoids induced by reprogramming compounds may be associated with the MAPK signaling pathway, TGF-beta signaling pathway, PI3K/Akt signaling pathway, and so on. MAPK is a widely expressed and evolutionarily conserved protein that plays a crucial role in mammalian sex determination through the signaling cascade involving GADD45G - p38 MAPK - GATA4 - SRY 66-68. Mutations in the MAP3K4 (a MAPK kinase) gene interrupt the expression of the SRY, leading to the failure to activate the testicular determination gene expression pathway and, consequently, to the inability to achieve Sertoli cell differentiation and form testicular cords 69. The TGF-β superfamily comprises activins, inhibins, BMPs, growth and differentiation factors (GDFs), and TGF-β homodimer proteins. The TGF-β/Smad signaling pathway is essential for regulating the proliferation and lactate supply of Sertoli cells 70, 71. Furthermore, TGF-β is involved in the regulation of both the proliferation and differentiation of Stem Leydig cells into testosterone-producing Leydig cells 72. The PI3K/Akt signaling pathway is a well-established regulator of cell metabolism, proliferation, and survival 73. IGF stimulates PI3K/Akt signaling and enhances FSH-FSHR activity in an autocrine manner, thereby regulating the expression of HSD3B, α-inhibin, STAR, CYP19, and LHCGR 74. The target genes regulated by reprogramming compounds constitute a complex regulatory network that ultimately orchestrates the regulation of intricate cell associations and intercellular signals in testicular organoids.

Additionally, a significant advantage of our organoids was the formation of a functional BTB, which is facilitated by tight junctions between mature Sertoli cells, preventing the ingress of harmful molecules into the seminiferous tubules 75. Interestingly, we found that *CLDN11*, a key marker for tight junction formation, was consistently lower in monolayer cultures but showed significant induction in 3D cultures, suggesting that *CLDN11* expression requires a more complex tissue structure or additional factors. We also assessed whether hiPSC-derived Sertoli-like cells were sufficient to form a complete tight junction at the protein level. The presence of CLDN11 and ZO-1 at the protein level confirmed that Sertoli-like cells in our organoids are maturing and establishing cell-to-cell connections akin to natural human Sertoli cells, thereby forming a BTB. However, *in vivo*, Sertoli cells mature and establish functional and complete BTB during puberty, which may differ from the timing of BTB formation in testicular organoids 76. We speculated that this phenomenon could be attributed to the activation of specific signaling pathways or cell-cell interactions in the *in vitro* culture environment, prompting the relatively primitive Sertoli-like cells in the organoids to initiate some programs related to BTB formation in advance, as observed in our study. However, we also recognize that there may be a certain gap between this early-formed structure and the mature BTB *in vivo*, and further optimization of culture conditions is necessary to develop testicular organoids that fully mimic the characteristics of the mature BTB. Additionally, the functionality of hormone receptors within the organoids is crucial for testicular function. Our analysis of LHCGR, FSHR, and AR expression in organoids at various stages, compared with testicular tissue from mice at different developmental stages, revealed a gradual decline in receptor expression, mirroring *in vivo* trends (data are shown in Figure S5). This suggests that the receptor development process in testicular organoids parallels that occurring *in vivo*. The Leydig cells within the organoids demonstrated endocrine functionality and responsiveness to LH. Furthermore, FSH was shown to enhance the levels of INHBB and lactate in Sertoli cells. Collectively, these data demonstrated that our organoids were responsive to gonadotropins and exhibited responsive characteristics to HPG axis regulation under *in vitro* culture conditions, indicating that they have certain similarities to *in vivo* testicular tissue. This highlights their potential as a reliable model for studying testicular physiology and related hormonal interactions. In the future, the reimplantation of testicular organoids *in vivo* may provide important insights into the overall function of the testis and the regulation of hormones at the systemic level in a more physiologically relevant context.

To truly mimic the testicular microenvironment and simulate spermatogenesis *in vitro*, integration of primordial germ cells (PGCs) is required. It is worth noting that, several studies have successfully generated human primordial germ cell-like cells (hPGCLCs) from hPSCs *in vitro* 77, 78. The hPGCLCs in these studies exhibited transcriptome profiles akin to those of early hPGCs and demonstrated potential for epigenetic reprogramming. However, the absence of a testicular microenvironment presents significant challenges in generating mature gametes with meiotic competence from hPGCLCs and in recapitulating spermatogenesis. At six weeks postfertilization, one of the earliest morphological changes observed in the male gonad is the formation of nascent “cord-like” structures composed of PGCs and Sertoli lineage cells, surrounded by fetal Leydig and interstitial cells. In humans, this fundamental niche structure persists throughout the fetal and postnatal stages, while the organized formation of seminiferous tubules occurs only during pubertal development 79. To confirm the developmental stage of the testicular organoids, we conducted an integrated analysis of the testicular organoid scRNA-seq data alongside a previously published dataset from human embryonic and fetal testicular tissue at 6, 8, and 16 weeks postfertilization (GSE143356) 31. The results indicated that the testicular organoids exhibited a similar proportion of specific cell types compared to those observed in the week 8 embryonic testicular tissue. This similarity suggests that the testicular organoids we constructed can effectively mimic the testicular niche at the 8-week postfertilization stage, exhibiting potential for simulating the early testicular structure and the testicular somatic cell microenvironment. Such characteristics are crucial for understanding the early stages of testicular development and for utilizing testicular organoids to support the meiosis of PGCs and the formation of gametes, as well as for drug evaluation purposes. While the testicular organoids showed significant similarities to the 8-week embryonic gonad, we also acknowledged some key differences. For example, the testicular organoids had not yet formed the organized seminiferous tubules that characterized later stages of development. This gap highlighted the need for further optimization of our culture conditions to promote the formation of more complex structures, which would be a focus of our future research.

To validate the reliability of testicular organoids as a drug screening and evaluation model, we exposed the testicular organoids to three known reproductive toxicity: CdCl_2_, etoposide, and doxorubicin. We found that testicular organoids exhibited dose-dependent responses to drug treatments leading to reduced organoid viability and compromised BTB integrity, consistent with previous findings *in vivo* 80. This suggested that our organoids were responsive to drug treatment and could be used as drug screening tools to bridge the gap between 2D culture systems and animal models. Next, we investigated whether these organoids could serve as a model for assessing the effects of semaglutide on testicular function. Semaglutide is a GLP-1 receptor agonist (GLP-1 RA) that was approved by the FDA in 2021 as an effective weight loss drug for overweight or obese adults, but its effects on testicular function have also attracted attention 21, 81, 82. Bolze *et al.* found that normal-weight rats treated with GLP-1/glucagon receptor co-agonists experienced significant weight loss, which resulted in decreased serum levels of LH and testosterone 83. In an *in vivo* study involving 9 healthy adult men, intravenous infusion of GLP-1 following oral glucose administration was found to reduce testosterone pulses by 50% 84. A cohort study utilizing the TriNetX research network examined 3094 obese men aged 18-50 years who were treated with semaglutide and did not have diabetes, matching them with an equal number of controls 85. This study revealed that 1.53% of patients were diagnosed with testosterone deficiency, presenting a risk 1.9 times greater than that of the matched control group. However, the lack of humanized models limits the validation of these findings. In our study, using testicular organoids for *in vitro* assays, we demonstrated that semaglutide significantly reduced testosterone secretion in testicular organoids by inhibiting the expression of LH receptor (LHCGR) and downregulating the expression of steroidogenesis-related enzymes CYP17A1 and HSD17B3. Furthermore, semaglutide did not affect the expression of Sertoli cell receptors FSHR and AR, nor did it impact the BTB function in testicular organoids. Semaglutide may enter the testicular organoids by binding to specific drug transporters or receptors on Sertoli cells and then crossing the BTB through receptor-mediated endocytosis. The semaglutide then impacts the classical androgen pathway between adjacent Sertoli cells by reducing the interaction between testosterone and AR, thereby reducing the level of INHBB. However, the specific details of the mechanisms by which semaglutide affects the reproductive system remain unclear. Our testicular organoids provide a potential model for future investigations into the effect of semaglutide on fertility. Compared to traditional animal models, testicular organoids provided a more accurate assessment of the direct effects of semaglutide on testicular tissues without considering the interference of other organs. This suggests that they may serve as a valuable platform for elucidating the mechanisms by which semaglutide inhibits INHBB secretion, as well as for facilitating rapid and personalized modeling for male infertility and drug screening. However, the limitations of organoids in fully replicating the complex signaling pathways and cellular interactions within the testis may result in an incomplete evaluation of drug efficacy and safety.

Despite these advances, several unresolved issues and research directions warrant further exploration. The mechanism of action of small molecule compounds in inducing testicular lineage-directed differentiation remains elusive. Furthermore, while gonad-specific markers and organizational structure have been demonstrated, spermatogenesis has not been observed, limiting the organoids' utility in drug evaluation models. Future research should focus on verifying the clinical relevance of organoids as drug evaluation models. Additionally, current methods of organoid culture face challenges in fully replicating the intricate three-dimensional structure and cellular interactions found in the *in vivo* setting. The development of a dynamic culture system that can simulate blood flow and nutrient delivery *in vivo* may enhance the reconstruction of the complex structure of the testis.

In summary, our 3D testicular organoid model represents a significant advancement in the generation of human testicular tissue from human iPSCs, encompassing multiple testicular cell types, gene expression, hormone responsiveness, and BTB function. Importantly, in this study, we showed that the cellular composition and structural features of our testicular organoids provided an avenue for modeling the effects of semaglutide on testicular function, a challenge that has eluded existing model systems. The organoids offer a platform for deciphering the intricate mechanisms through which this drug exerts its influence, a realm that has remained largely uncharted due to the absence of pertinent human *in vitro* models. Our testicular organoids, therefore, offer a promising foundation for future research in reproductive biology and pharmacology, with the potential to become a cornerstone in understanding drug effects on testicular function and developing new therapeutic strategies.

## Conclusions

In this study, we established a method to generate testicular organoids from human iPSCs. Small molecule compounds were used for differentiation, and a combination of hanging drop and rotational culture systems for assembly. These organoids closely emulated human testicular tissue, exhibiting testicular cord-like structures and a functional blood-testis barrier. RNA sequencing and functional assays validated the proportion of testicular somatic cells, as well as their gene expression and endocrine functions similar to in-vivo testicular tissue. Notably, these organoids displayed sensitivity to semaglutide. Treatment with semaglutide resulted in reduced testosterone levels and downregulation of *INHBB* expression, highlighting their potential as valuable models for studying testicular function, drug toxicity, and the effects of compounds like semaglutide on testicular health.

## Supplementary Material

Supplementary figures and tables.

## Abbreviations

AMH: anti-Müllerian hormone
AR: androgen receptor
BMI: body mass index
BMP4: bone morphogenetic protein 4
BTB: blood-testis barrier
cis-eQTL: cis-expression quantitative trait locus
DEGs: differentially expressed genes
FGF 9: fibroblast growth factor 9
FSH: follicle-stimulating hormone
GCs: germ cells
GLP-1 RA: glucagon-like peptide-1 receptor agonist
GWAS: genome-wide association study
hESCs: human embryonic stem cells
hiPSCs: human induced pluripotent stem cells
hPGCLCs: human primordial germ cell-like cells
hPSCs: human pluripotent stem cells
HTT: human testicular tissue
IM: intermediate mesoderm
INHBB: inhibin B
IV: instrumental variable
IVW: inverse variance weighted
LCs: Leydig cells
LH: luteinizing hormone
MR: mendelian randomization
PGCs: primordial germ cells
PS: primitive streak
PTMs: peritubular myoid cells
SCs: Sertoli cells
SNPs: single nucleotide polymorphisms
SSCs: spermatogonial stem cells
TJ: tight junction
TOs: testicular organoids
UMAP: uniform manifold approximation projection
