# Supplementary material for: Construction of human pluripotent stem cell-derived testicular organoids and their use as humanized testis models for evaluating the effects of semaglutide
Source: Theranostics. 2025 Jan 27;15(6):2597–623. doi: 10.7150/thno.104523 (PMC11840739; doi:10.7150/thno.104523)
Supplement: Supplementary file 1 — Supplementary figures and tables. [file thnov15p2597s1.pdf]

Supplementary data

Construction of human pluripotent stem cell-derived testicular organoids and their use as humanized testis models for evaluating the effects of semaglutide

Rufei Huang[1#], Huan Xia[1#], Tao Meng[1#], Yufei Fan[1], Xun Tang[1], Yifang Li[1], Tiantian Zhang[1], Jingxian Deng[1], Bing Yao[2*], Yadong Huang[1,3,4*], Yan Yang[1,3,4*]

1. State Key Laboratory of Bioactive Molecules and Druggability Assessment, Guangdong Basic Research Center of Excellence for Natural Bioactive Molecules and Discovery of Innovative Drugs, College of Life Science and Technology, Jinan University, Guangzhou, 510632, China.

2. Department of Reproductive Medicine, Jinling Hospital, Affiliated Hospital of Medical School, Nanjing University, Nanjing, 210002, China.

3. National Engineering Research Center of Genetic Medicine, Guangzhou, 510632, China.

4. Guangdong Province Key Laboratory of Bioengineering Medicine, Guangzhou, 510632, China.

# These authors contributed equally to this work.

* Corresponding author: Yan Yang (yangyan107@jnu.edu.cn), Yadong Huang (tydhuang@jun.edu.cn), Bing Yao (yaobing@nju.edu.cn).

This file includes: Figures S1 to S12 and Tables S1 to S20

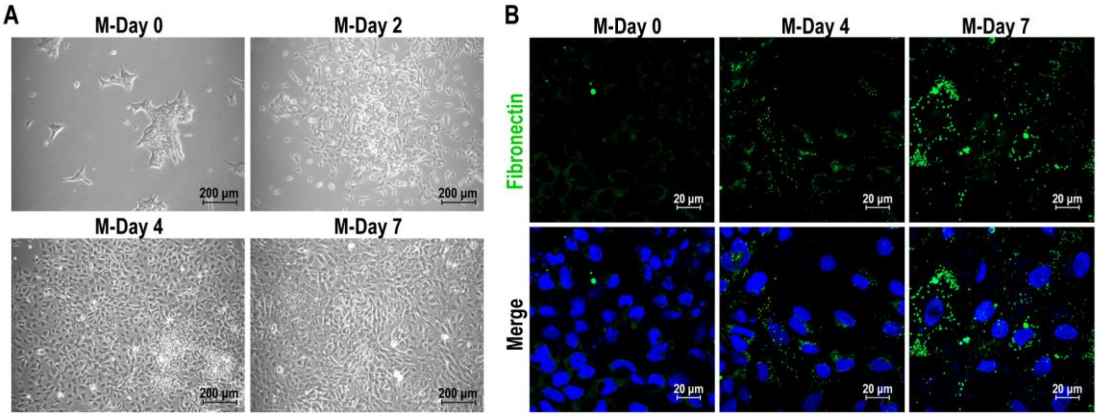

Figure S1. Conversion of hiPSCs into pre-testis-like cells by a combination of several small molecules, related to Figure 1.

(A) The cell morphology changes from pluripotent stem cell morphology to testicular cell morphology during the induction process. Representative images at days 0, 2, 4, and 7 were shown. Scale bar, 200 μm. **(B)** Immunofluorescence analysis was conducted at days 0, 4, and 7 during cell differentiation. The basement membrane was labeled with Fibronectin. Nuclei were counterstained with DAPI (blue). Scale bar, 20 μm.

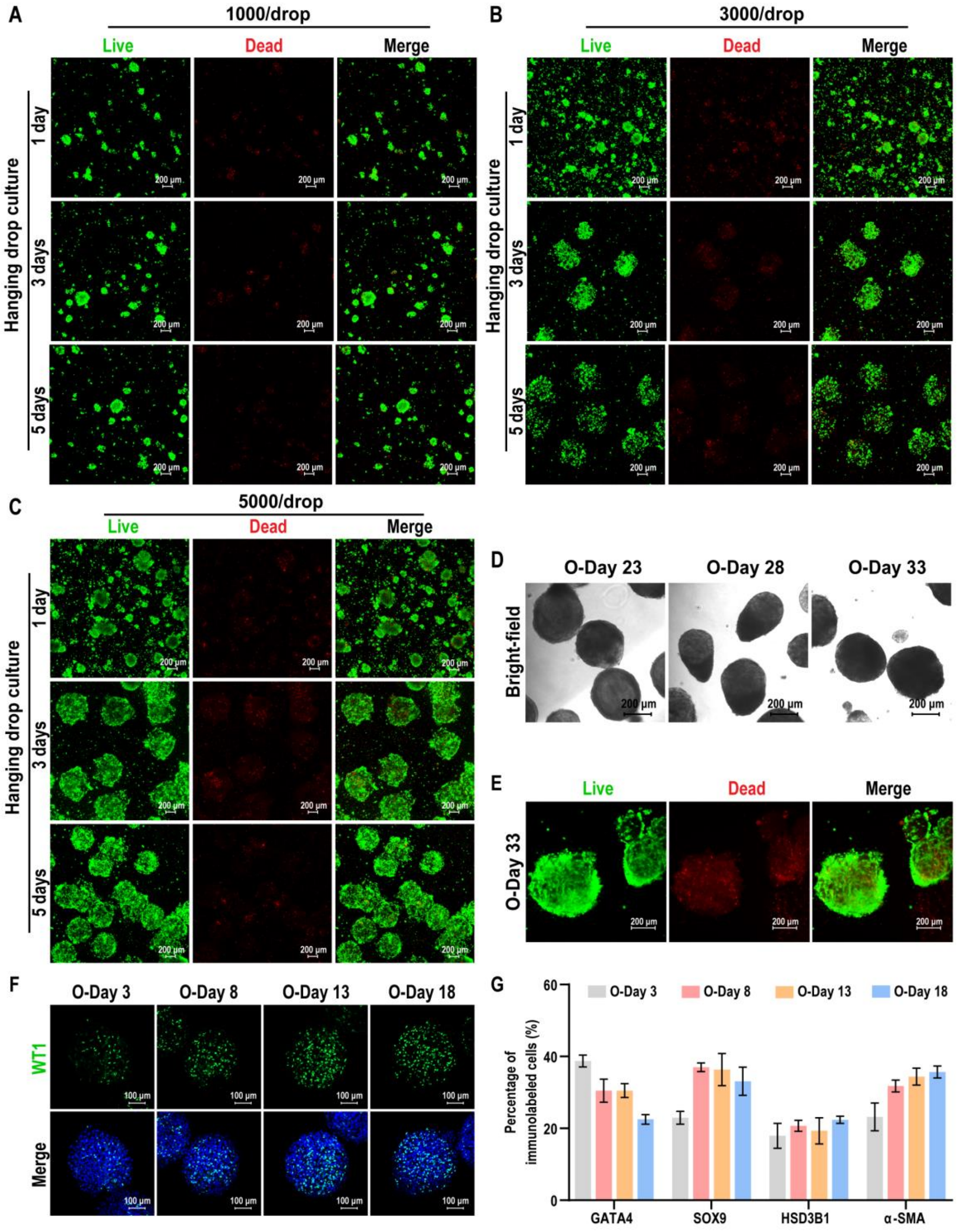

Figure S2. Preparation of testicular organoids, related to Figure 2.

(A-C) Cell spheroids of 1000, 3000, and 5000 cells/drop were stained at days 1, 3, and 5 of hanging drop culture using a LIVE/DEAD Cell Imaging Kit (live cell = green, dead cell = red). Scale bar, 200 μm. **(D)** Bright-field images of cell spheroids

(5000 cells/drop) after 23, 28, and 33 days of organoid culture. Scale bar, 200 μm. **(E)** Live/Dead staining images of cell spheroids (5000 cells/drop) at day 33 of organoid culture. Scale bar, 200 μm. **(F)** Immunofluorescence analysis was conducted on days 3, 8, 13, and 18 organoids. Bipotential gonad cells were identified by the marker WT1. Nuclei were counterstained with DAPI (blue). The scale bar represents 100 μm. **(G)** The percentage of bipotential gonad cells (GATA4), Sertoli cells (SOX9), Leydig cells (HSD3B1), and peritubular myoid cells (α-SMA) in day 3, 8, 13, and 18 organoids staining in Figure 2J-M. Images were obtained from three technical replicates, and the data are presented as the mean ± SD.

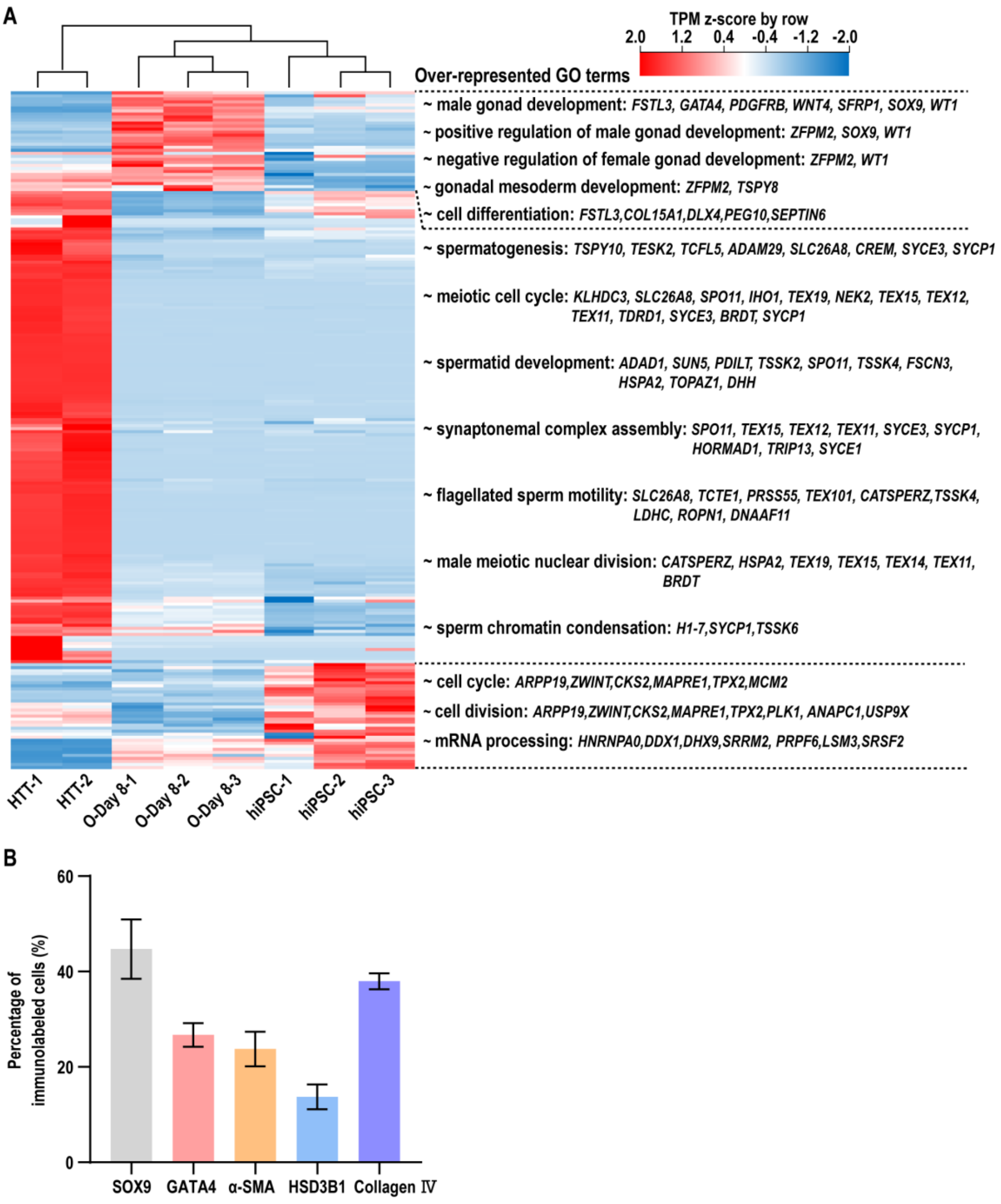

Figure S3. Transcriptome analysis and characterization of testicular organoids, related to Figure 3.

(A) Cluster heat map of 656 testis-specific genes in hiPSC, O-Day 8, and HTT. **(B)** The percentage of immunolabeled-positive cells in day 8 organoid staining in Figure 3F, was quantified for n=5 (GATA4, α-SMA, HSD3B1, Collagen IV) or n=20 images (SOX9) from technical replicates using imageJ, presented as mean ± SD.

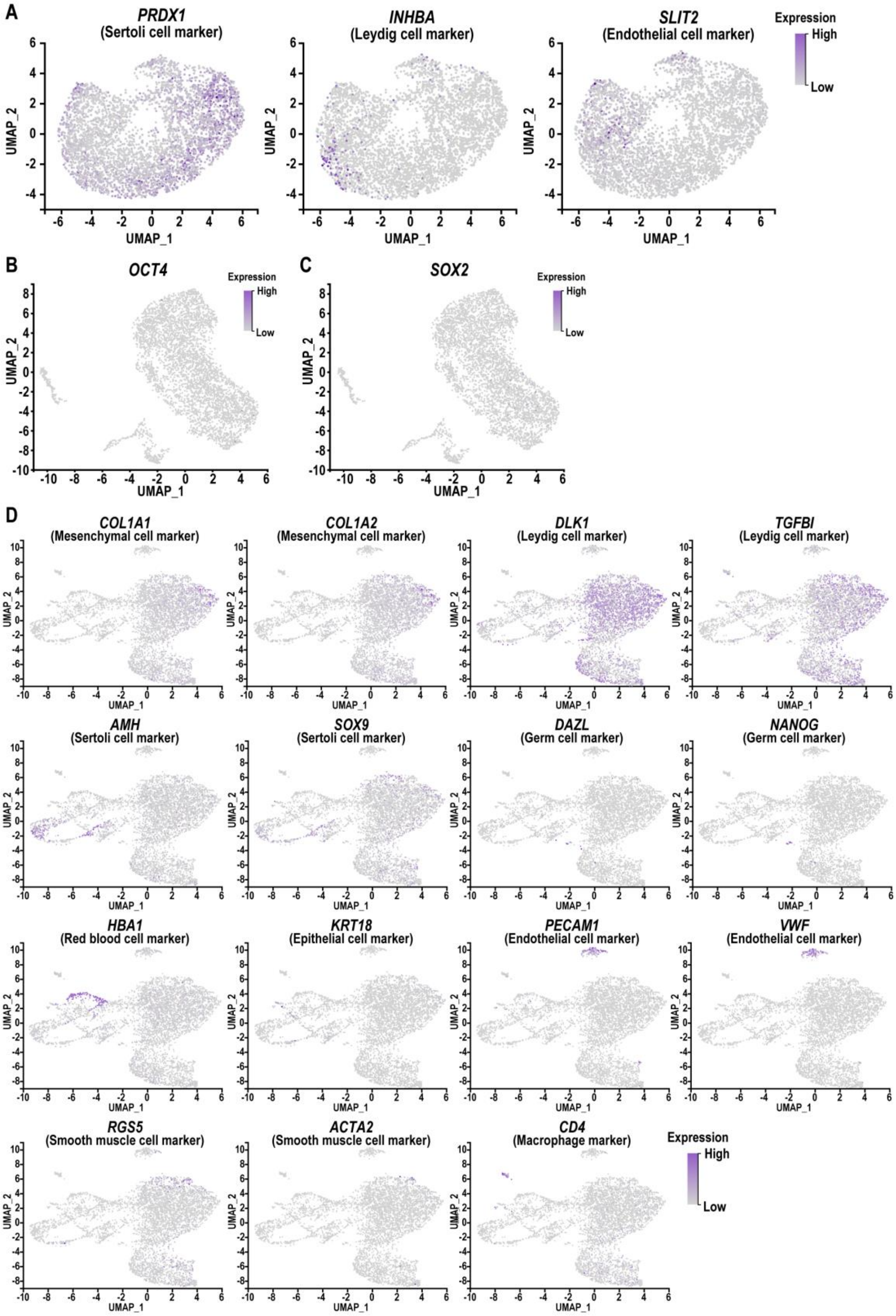

Figure S4. scRNA-seq reveals cell-type heterogeneity in testicular organoids, related to Figure 4.

(A) Expression patterns of specific marker genes in different cell types projected on the UMAP plot in Figure 4C. **(B-C)** Feature plots for the expression of pluripotency marker genes (*OCT4* and *SOX2*) in different cell clusters. **(D)** Expression patterns of specific marker genes in different cell types projected on the UMAP plot in Figure 4G. The shades of purple in the UMAP plot reflect the relative expression levels of the corresponding genes.

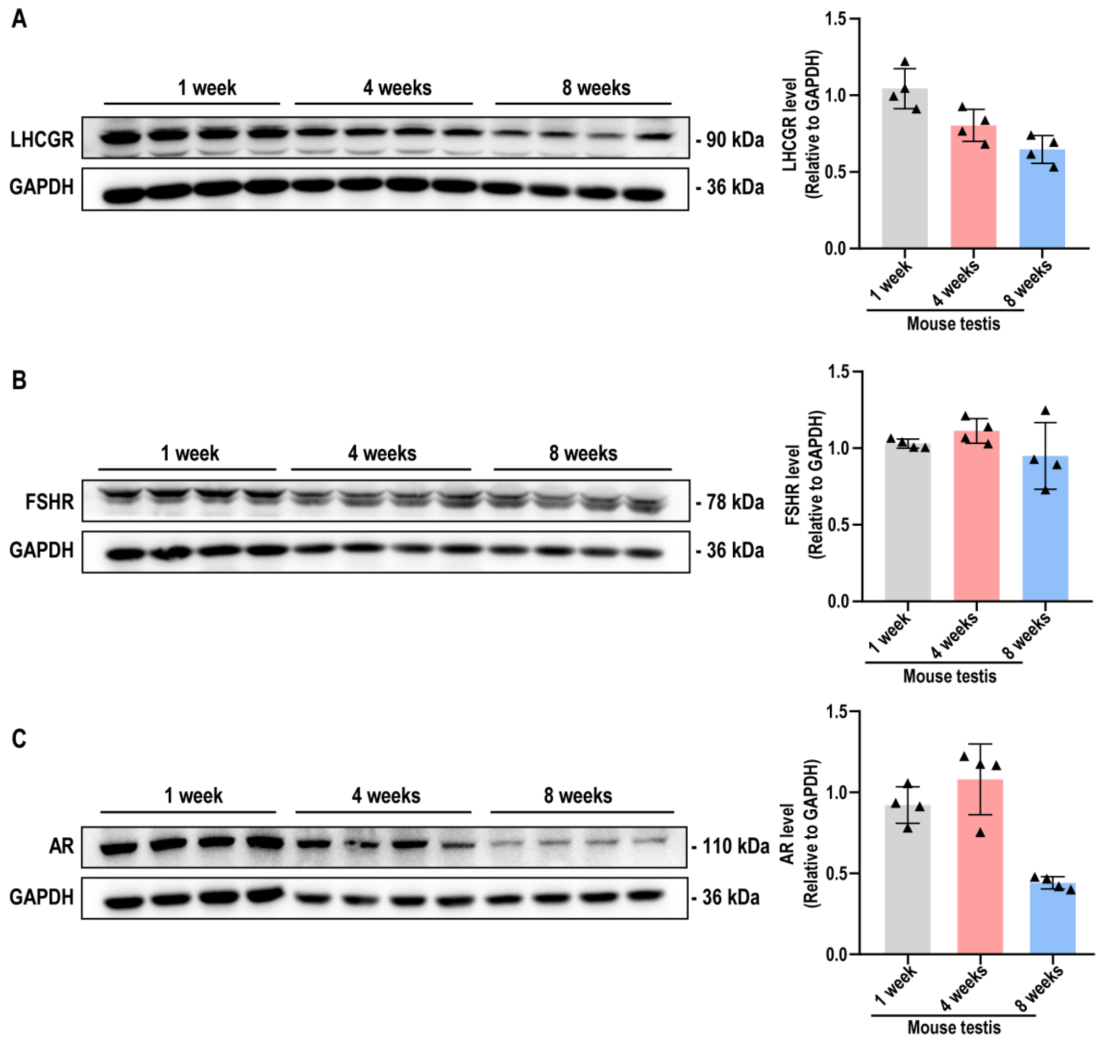

Figure S5. Expression of hormone receptor proteins in testicular tissue of mice at different ages, related to Figure 5.

(A-C) Protein expression of LHCGR, FSHR, and AR in testicular tissue of mice at 1, 4, and 8 weeks of age were analyzed by Western blot. GAPDH served as the loading control. Data are expressed as mean ± SD (n = 4 per group).

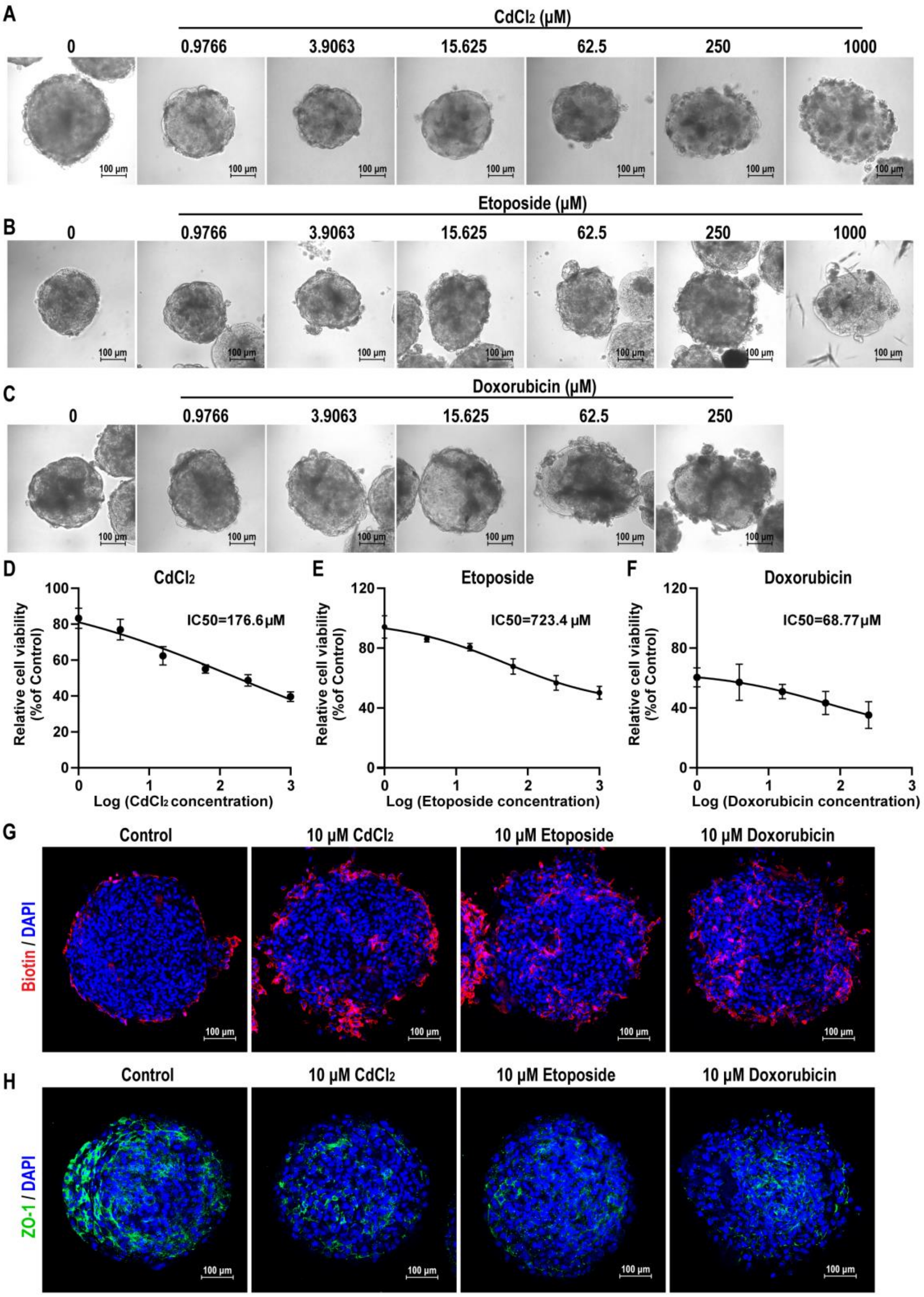

Figure S6. Assessment of cell viability and BTB integrity of testicular organoids after compound treatment, related to Figure 6

(A-C) Representative bright-field images showed organoids after 24 h of exposure to

different concentrations of $CdCl_2$, etoposide, and doxorubicin (0, 0.9766, 3.9063, 15.625, 62.5, 250, and 1000 μM). Scale bar, 100 μm. **(D-F)** Dose-response curves were generated using CCK8 assay to calculate IC50 values of $CdCl_2$, etoposide, and doxorubicin on the organoids. These data were obtained from three independent experiments, and presented as mean ± SD. **(G)** After treating day 8 organoids with 10 μM $CdCl_2$, etoposide, and doxorubicin for 24 h, the BTB integrity was evaluated using the sulfo-NHS-LC-biotin assay. **(H)** Immunofluorescence was employed to detect ZO-1 protein expression in day 8 organoids exposed to 10 μM $CdCl_2$, etoposide, and doxorubicin for 24 h. Nuclei were counterstained with DAPI (blue). Scale bar, 100 μm.

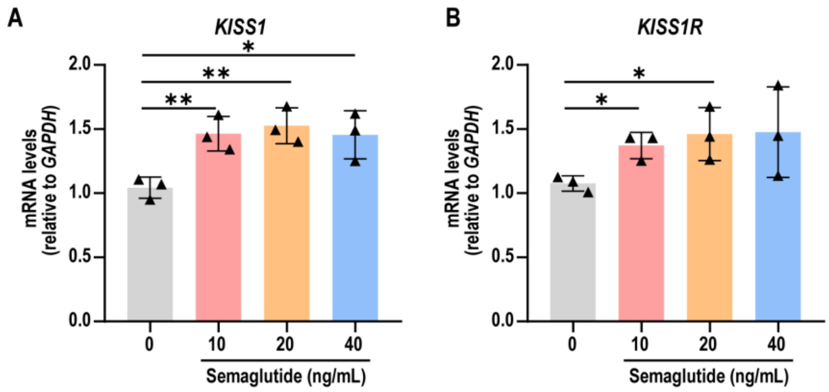

Figure S7. Detection of the expression of GLP-1R downstream markers in testicular organoids after semaglutide treatment, related to Figure 7.

(A-B) The expression level of *KISS1* and *KISS1R* in organoids treated with semaglutide (0, 10, 20, and 40 ng/mL) for 24 h was detected by qPCR. The mRNA copy number of each gene was normalized with *GAPDH*. All data were from three independent experiments and were expressed as mean ± SD, $^{*}P < 0.05$; $^{**}P < 0.01$.

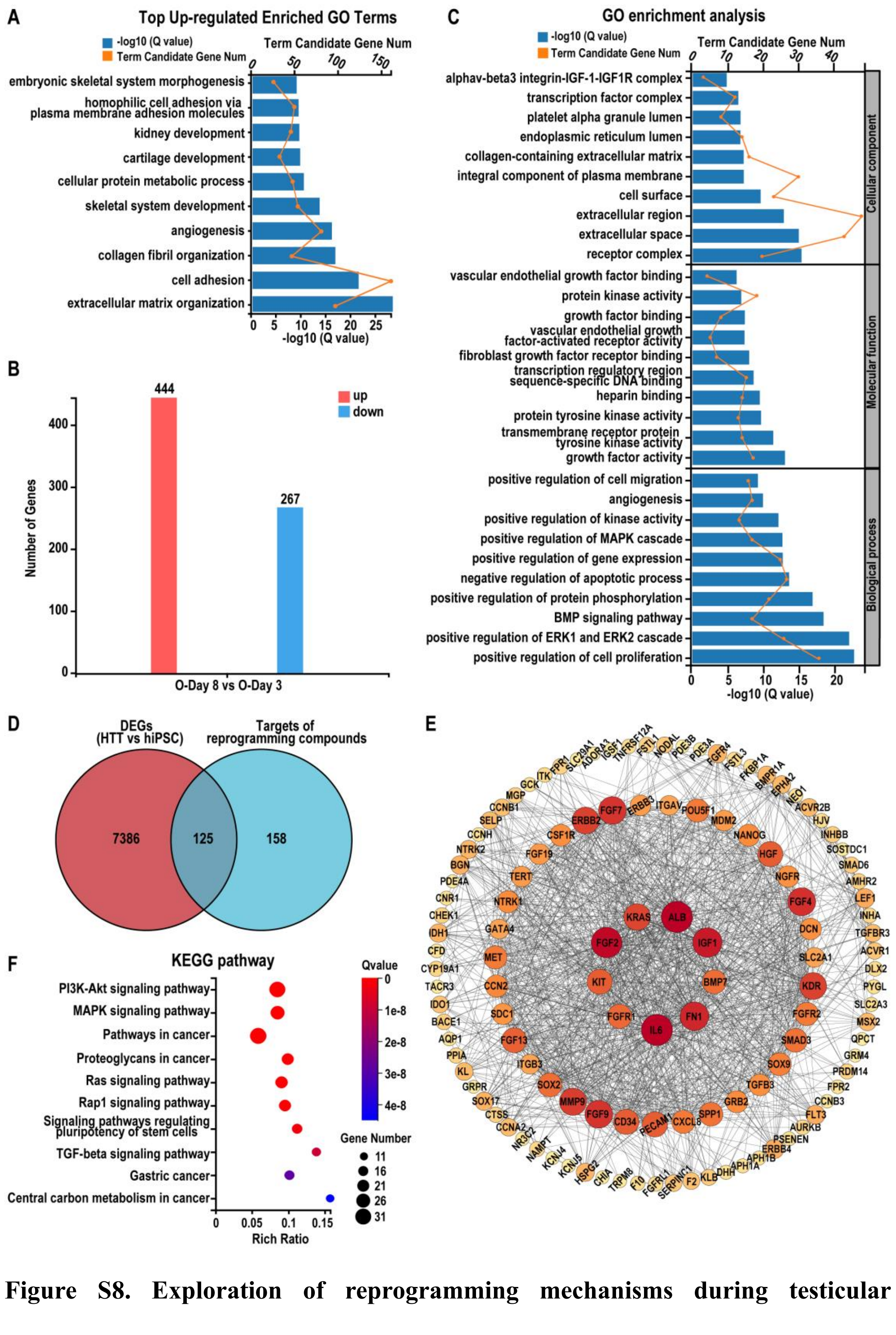

Figure S8. Exploration of reprogramming mechanisms during testicular organoid induction, related to Figure 8.

(A) Analysis of GO terms for the top ten GO categories of up-regulated genes in day

3 organoids compared to hiPSCs (|$\log_2$FoldChange| ≥ 2, Q value ≤ 0.01). **(B)** Number of up- or down-regulated genes between day 3 and day 8 organoids (|$\log_2$FoldChange| ≥ 2, Q value ≤ 0.01). **(C)** GO enrichment analysis of 109 common targets (the top ten results of cellular component, molecular function, and biological process enrichment analysis respectively). **(D)** The Venn diagram analysis of the DEGs (HTT vs hiPSC) and the predicted targets of reprogramming compounds (CHIR, Activin A, BMP4, FGF9, and heparin). **(E)** The PPI network of 125 intersection genes in Figure S8D. **(F)** The KEGG enrichment analyses of 125 intersection genes in Figure S8D (the top ten results).

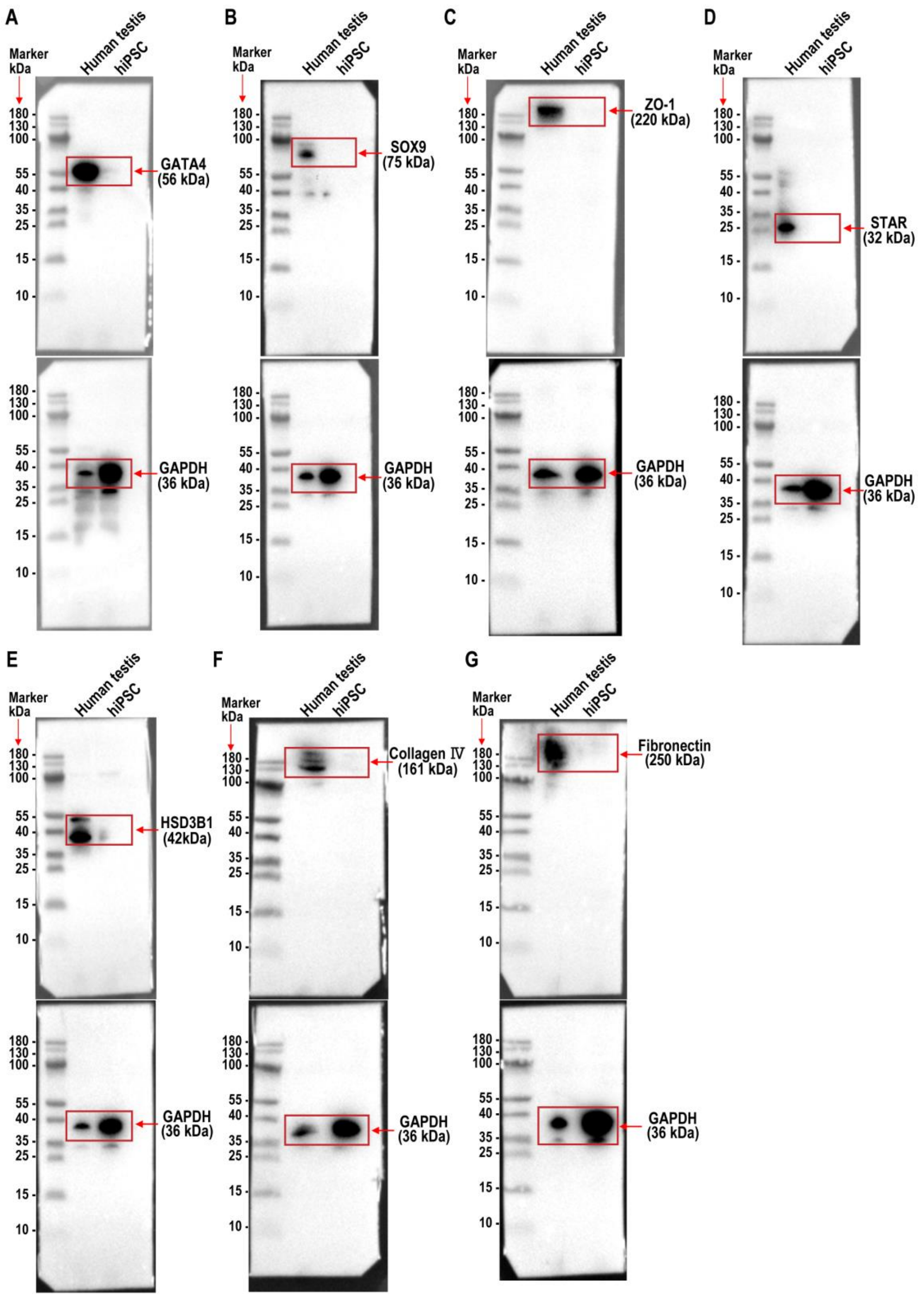

Figure S9. Full-size Western blots demonstrate the specificity of the antibodies used in Figure 1.

(A-G) Antibody specificity for (A) GATA4, (B) SOX9, (C) ZO-1, (D) STAR, (E)

HSD3B1, (F) Collagen IV, and (G) Fibronectin was verified with human testicular tissue (as positive control) and hiPSC (as negative control). GAPDH served as the loading control.

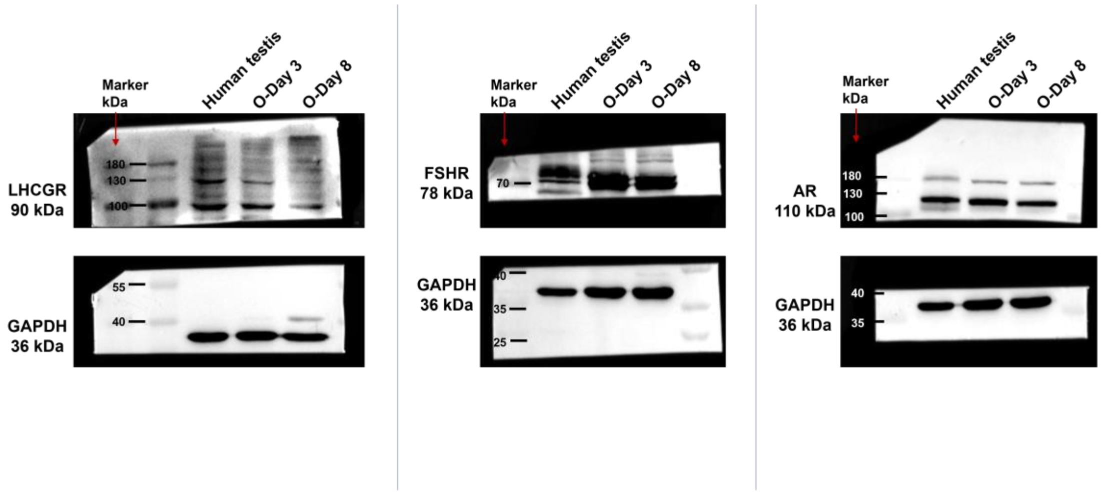

Figure S10. Full-size western blotting images of Figure 5B.

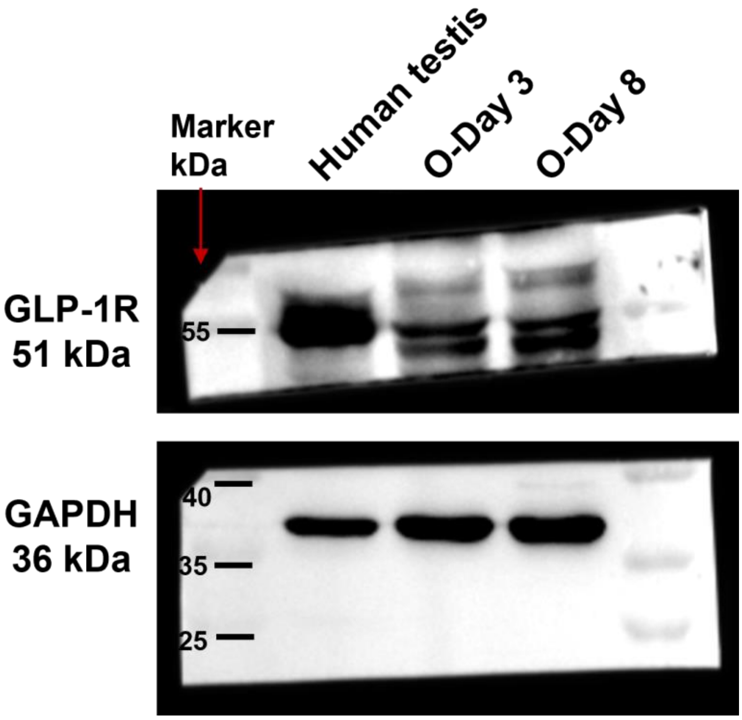

Figure S11. Full-size western blotting images of Figure 6B.

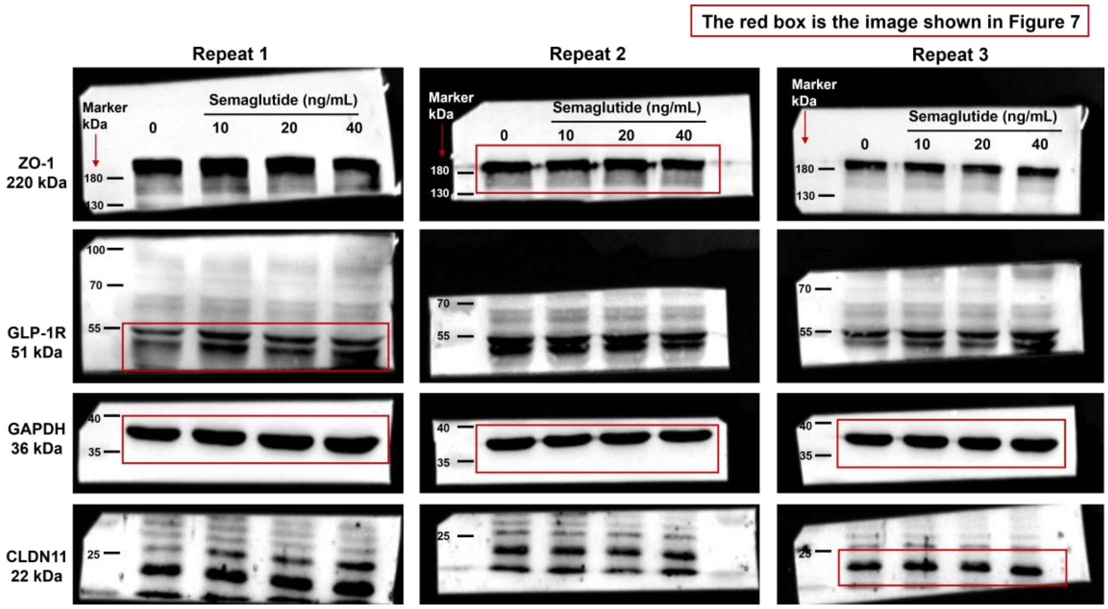

Figure S12. Full-size western blotting images of Figure 7B and 7O.

Table S1. Primer sequences used in qRT-PCR analysis.

Gene	Forward primer	Reverse primer
GAPDH	AGCCACATCGCTCAGACAC	GCCCAATACGACCAAATCC
OCT4	AGCAAAACCCGGAGGAGT	CCACATCGGCCTGTGTATATC
SOX2	TGAATCAGTCTGCCGAGAATCC	TCTCAAACTGTGCATAATGGAGT
TBXT	AGGTACCCAACCCTGAGGA	GCAGGTGAGTTGTCAGAATAGGT
LHX1	ATGCAACCTGACCGAGAAGT	CAGGTCGCTAGGGGAGATG
PAX2	GCAACCCCGCCTTACTAAT	AACTAGTGGCGGTCATAGGC
WT1	GAATGGACAGAAGGGCAGA	GACACCGTGCGTGTGTATTC
GATA4	CTGTCATCTCACTACGGGCA	GGAGACGCATAGCCTTGT
LHX9	CTTACTTCAACGGTACGGGC	GGTGGATAAGGCTGCTGGT
NR0B1	CAAGGAGTACGCCTACCTCA	GCGTCATCCTGGTGTGTTC
HSD3B2	CTTGGACAAGGCCTTCAGAC	GGCTCATCCAGAATGTCTCC
GADD45G	ACTAGCTGCTGGTTGATCGC	CAACTCATGCAGCGCTTTC
ZFPM2	CTTGGCAAGGAGTGGAAGAC	TCTTCACCCTCAGAGATGGC
EMX2	CTCAGCCTCACGGAAACTCA	TTGCGAATCTGAGCCTTCTT
AMH	GCTACCTGGTGTTAGCGGTG	GTACTCAGCCGGGAGTCCTCT
DHH	AACCCCGACATCATCTTCAA	ACATGTTCATCACGGCAATG
SOX9	CCGAAAGCGGAGCTCGAAAC	AGTTTCCGGGGTTGAAACTGG
FGF9	GTGGACTCTACCTCGGGATG	CCAGTTTTCTTCGAACTGTTCTC
CLDN11	GGCTGGTGTTTTGCTCATTCT	AGCACCAATCCAGCCTGCATA
HSD3B1	CCTTCGGACCAGAATTGAGA	ATACAGGCGGTGTGGATGAT
CYP17A1	TATGGCCCCATCTATTCGGTT	GCGATACCCTTACGGTTGTTG
HSD17B3	GTCAACAATGTCGGAATGCTTC	TGATGTTACAATGGATGAGGCTC
FSHR	GGATCCATTCCCTGCCT	ATGAAGCTCAGAGATTTGCC
GDNF	GACCCCAGGATTGCGAACT	TCTGGCATATTTGAGTCACTGC
STAR	TAGCGACATTCAAGCTGTGC	GTTCAGCTCCTGGCTGATG
INHBB	GCGCGTTTCCGAAATCATCA	GGACGTAGGGCAGGAGTTTC

BMP4	CGGAAGCTAGGTGAGTGTGG	TCGAGATAGCTTGGACGGGA
SCF	GCCAGCTCCCTTAGGAATGAC	CTGCCCTTGTAAGACTTGGCT
CXCL12	GAAAGCCATGTTGCCAGAGC	GCGTCTGACCCTCTCACATC
AR	GACATGCGTTTGGAGACTGC	TTTCTTCAGCTTCCGGGCTC
LHCGR	CACATAACCACCATACCAGGAAA	AAGTCAGTGTCGTCCCATTGA
CYP11A1	GCAGTGTCTCGGGACTTCG	GGCAAAGCGGAACAGGTCA
KISS1	CACTTTGGGGAGCCATTAGA	CAGTAGCAGCTGGCTTCCTC
KISS1R	GGACGTGACCTTCCTCCTGT	GTACCAGCGGTCCACACTCA

Table S2. Primary and secondary antibodies used in the experiments.

Antibodies	Source	Identifier
GATA4	Abcam	Cat# ab307823; RRID: AB_3105880
SOX9	Abcam	Cat# ab185966; RRID: AB_2728660
ZO-1	Abcam	Cat# ab221547; RRID: AB_2892660
STAR	Proteintech	Cat# 12225-1-AP; RRID: AB_2115832
HSD3B1	Abcam	Cat# ab167417; RRID: AB_3107063
Collagen IV	Abcam	Cat# ab6586; RRID: AB_305584
Fibronectin	Affinity Biosciences	Cat# AF0738; RRID: AB_2834137
α-SMA	Abcam	Cat# ab32575; RRID: AB_722538
WT1	Abcam	Cat# ab89901; RRID: AB_2043201
Caspase 9	Proteintech	Cat# 66169-1-Ig; RRID: AB_2833257
Claudin 11	Thermo Fisher Scientific	Cat# 36-4500; RRID: AB_2533259
LHCGR	Proteintech	Cat# 19968-1-AP; RRID: AB_10697685
FSHR	Abcam	Cat# ab103874; RRID: AB_10899034
AR	Proteintech	Cat# 22089-1-AP; RRID: AB_11182176
GLP-1R	Bioss	Cat# bs-1559R; RRID: AB_10857357
Goat Anti-Rabbit IgG H&L (Alexa Fluor® 488)	Abcam	Cat# ab150077; RRID: AB_2630356

Table S3. IVs of GLP-1R for the drug target mendelian randomization.

SNP	P value	A1	A2	sample size	EAF	BETA	SE	F
rs1678701	3.45E-13	A	G	29498	0.424	-0.061	0.00832	52.925
rs1678697	4.34E-13	G	A	29502	0.425	-0.060	0.00832	52.478
rs1678696	4.37E-13	G	A	29503	0.425	-0.060	0.00832	52.463
rs1629877	4.82E-13	C	A	29504	0.425	-0.060	0.00832	52.274
rs1678700	5.39E-13	G	A	29504	0.424	-0.060	0.00832	52.051
rs1678702	5.44E-13	C	A	29504	0.424	-0.060	0.00832	52.035
rs1678695	5.58E-13	A	G	29504	0.425	-0.060	0.00832	51.983
rs1738203	6.38E-13	A	G	29507	0.568	-0.060	0.00830	51.723
rs1678707	6.49E-13	G	A	29506	0.425	-0.060	0.00832	51.688
rs1678706	6.83E-13	G	A	29506	0.425	-0.060	0.00832	51.588
rs9380787	7.07E-13	G	T	29616	0.557	-0.059	0.00826	51.517
rs6919465	7.11E-13	G	A	29506	0.425	-0.060	0.00832	51.510
rs1678712	7.24E-13	G	A	29506	0.425	-0.060	0.00832	51.473
rs984524	7.43E-13	G	A	29506	0.425	-0.060	0.00832	51.420
rs1738199	7.61E-13	A	G	29505	0.565	-0.059	0.00830	51.375
rs1738212	7.8E-13	C	A	29506	0.425	-0.060	0.00832	51.327
rs1678693	1.04E-12	C	T	29506	0.425	-0.059	0.00832	50.762
rs1678691	2.06E-12	C	T	29506	0.426	-0.058	0.00832	49.423
rs9349100	2.36E-12	T	C	29507	0.564	-0.058	0.00829	49.151
rs1678692	2.5E-12	C	T	29508	0.426	-0.058	0.00832	49.043
rs9394544	3.05E-12	A	G	29500	0.569	-0.058	0.00831	48.654
rs1678682	6.45E-12	G	A	29508	0.566	-0.057	0.00830	47.183

IVs: Instrumental variables; SNP: single nucleotide polymorphism; A1: effect allele; A2: other allele; EAF: effect allele frequency; BETA: beta-coefficient; SE: standard error; F: F statistic.

Table S4. Detailed information of outcome data in this study.

phenotype	Consortium	GWAS ID	PMID	Sample size	Sex	Web Source
Orchitis and epididymitis	FinnGen	N14_ORHCEPINDIDYMITIS	NA	2688 in case and 119,297 in control	Males	https://storage.googleapis.com/finngen-public-data-r9/summary_stats/finngen_R9_N14_ORHCEPINDIDYMITIS.gz
Luteinizing hormone levels	NA	GCST90241825	29875488	3301	Males and Females	https://www.ebi.ac.uk/gwas/studies/GCST90241825
Follicle-stimulating hormone	NA	GCST90012661	33441150	3484	Males and Females	https://www.ebi.ac.uk/gwas/studies/GCST90012661
Bioavailable testosterone	NA	ieu-b-4868	NA	184,205	Males	https://gwas.mrcieu.ac.uk/datasets/ieu-b-4868/
Free testosterone levels	NA	GCST90239825	36653534	161,887	Males	https://www.ebi.ac.uk/gwas/studies/GCST90239825
Inhibin beta A chain: Inhibin beta B chain heterodimer	NA	prot-a-1555	29875488	3301	Males and Females	https://gwas.mrcieu.ac.uk/datasets/prot-a-1555/
HbA1c levels	NA	ebi-a-GCST90014006	34017140	389,889	NA	https://gwas.mrcieu.ac.uk/datasets/ebi-a-GCST90014006/
Type 2 diabetes	NA	ebi-a-GCST006867	30054458	655,666	NA	https://gwas.mrcieu.ac.uk/datasets/ebi-a-GCST006867/
BMI	MRC-IEU	ukb-b-19953	NA	461,460	Males and Females	https://gwas.mrcieu.ac.uk/datasets/ukb-b-19953/

Table S5. Positive control analysis in IVW method.

Outcome	P value	OR	or_lci95	or_uci95	BETA	SE
HbA1c	4.8E-43	0.90	0.89	0.92	-0.1005	0.01614
Type 2 diabetes	1.9E-15	0.80	0.76	0.84	-0.2253	0.02834
BMI	0.0026	0.98	0.96	0.99	-0.0162	0.00715

IVW: Inverse variance weighted; OR: odds ratio; BETA: beta-coefficient; SE: standard error.

Table S6. Main result in IVW method.

Outcome	P value	OR	or_lci95	or_uci95	BETA	SE
Orchitis and epididymitis	1.57E-05	1.56	1.27	1.91	0.444	0.1027
Follicle-stimulating hormone	2.54E-19	0.45	0.38	0.54	-0.797	0.0887
Luteinizing hormone	8.08E-11	0.53	0.44	0.64	-0.629	0.0968
Inhibin beta A chain: Inhibin beta B chain heterodimer	0.00015	0.71	0.6	0.85	-0.353	0.1807
Bioavailable testosterone	0.00053	0.96	0.94	0.98	-0.041	0.0118
Free testosterone	0.00062	0.96	0.94	0.98	-0.043	0.0124

IVW: Inverse variance weighted; OR: odds ratio; BETA: beta-coefficient; SE: standard error.

Table S7. Sensitivity analyses results in the median weighted method.

Outcome	P value	OR	or_lci95	or_uci95	BETA	SE
Orchitis and epididymitis	8.17E-05	1.63	1.28	2.09	0.444	0.1027
Follicle-stimulating hormone	2.17E-12	0.45	0.36	0.57	-0.797	0.0887
Luteinizing hormone	6.35E-07	0.55	0.44	0.7	-0.629	0.0968
Inhibin beta A chain: Inhibin beta B chain heterodimer	1.19E-03	0.71	0.57	0.87	-0.348	0.1073
Bioavailable testosterone	7.09E-03	0.96	0.94	0.99	-0.041	0.0118
Free testosterone	5.72E-03	0.96	0.93	0.99	-0.043	0.0124

OR: odds ratio; BETA: beta-coefficient; SE: standard error.

Table S8. Heterogeneity analysis with Cochrane's Q statistic.

Outcome	method	Q	Q_df	Q_p value
Orchitis and epididymitis	IVW	0.498562455	21	1
Follicle-stimulating hormone	IVW	0.08895284	21	1
Luteinizing hormone	IVW	0.395449934	21	1
Inhibin beta A chain: Inhibin beta B chain heterodimer	IVW	0.412613544	21	1
Bioavailable testosterone	IVW	0.242100835	21	1
Free testosterone	IVW	0.09774217	21	1

IVW: Inverse variance weighted.

Table S9. Results of pleiotropy analysis.

Outcome	egger intercept	SE	P value
Orchitis and epididymitis	-0.2272	0.4119	0.5874
Follicle-stimulating hormone	-0.0087	0.3566	0.9807
Luteinizing hormone	-0.2270	0.3880	0.5650
Inhibin beta A chain: Inhibin beta B chain heterodimer	-0.0228	0.3566	0.9497
Bioavailable testosterone	-0.0033	0.0473	0.9452
Free testosterone	0.0017	0.0499	0.9729

SE: standard error.

Table S10. Drug targets of CHIR99021.

Database	Target symbol				
Swiss Target Prediction	CCNB3	CDK1	CCNB1	CCNB2	GSK3B
	GSK3A	BACE1	SLC29A1	ADORA3	PTGES
	CDK6	CCKBR	MAPK1	MAOA	HIF1A
	CHEK1	BLM	PYGL	PYGM	GCK
	PSEN2	PSENEN	NCSTN	APH1A	PSEN1
	APH1B	QPCTL	HCRTR2	HCRTR1	ADAMTS5
	CTSS	CSNK1D	PTPN1	TACR3	CDK9
	CCNT1	ERBB2	PIK3CD	PRKACA	AKT1
	CCNA2	CDK2	FPR1	CALCRL	TTK
	IL2	IDH1	PTAFR	TOP2A	ALOX5
	TRPV1	TNKS2	ITGAV	ITGB3	AURKB
	SLC2A1	MMP9	MMP1	MMP2	SLC2A3
	CFD	GRK2	AVPR2	AVPR1A	CCNE1
	EPHX2	MDM2	GPR119	TAAR1	TRPM8
	VDR	NR3C2	MAPKAPK2	AGTR1	PDE3A
	NTRK1	PDE3B	FPR2	CNR1	ITK
	QPCT	FKBP1A	MAP3K8	CYP26A1	CPT1A
	PDE4A	PDE4B	PDE4D	PDE4C	CYP19A1
	MKNK2	NOS2	TERT	BAD	MAPK11
	WEE1	LIMK2	NAMPT	PDE5A	FLT1
	PPIA	MAOB	GRM4	STK17B	BDKRB1
	RPS6KB1	IDO1	GRPR	F2	CDK7
	CCNH	ACHE			
ChEMBL	SCP2	AQP1	KCNJ5	ADH5	AKR1C4
	CHIA	KCNJ3	KCNJ4	ADH1A	ADH4
	ADH1B	ADH1C	ADH7	ADH6	

STITCH	GSK3B	GSK3A	BCL2L12	ENSG00000268643
TTD	GSK-3A	GSK-3B	CDK1	ERK2

Table S11. Drug targets of heparin.

Database	Target symbol				
ChEMBL	FGF4	PTN	HBEGF	FGF2	FGF1
	FGFR2	FGF7	KLB		
DrugBank	SERPINC1	F10	SELP	FGFR4	FGF4
	FGF19	FGFR1	FGF1	FGFR2	FGF2
	PF4	HGF			
TTD	EGFR	F2	F10	ATIII	FGF2
	FGF4	FGF1	HBEGF	FGF7	SERPIND1
	FGF8	FGFBP1	PTN	ALOX12B	

Table S12. Drug targets of activin A.

Database	Target symbol				
ChEMBL	ACVR1C	ACVR1B	INHBA	TGFBR1	ACVR2B
	ACVR1	ACVRL1	BMPR1A	ACVR2A	
TTD	TGFBR1	INHBA			
STRING	ACVR2A	FST	ACVR2B	ACVR1B	FSTL3
	INHBB	ACVR1C	INHA	TGFB3	TGFB1
	NODAL	IGSF1	SMAD3	LOC102723407	SMAD2
	BMPER				

Table S13. Targets of BMP4 from STRING database.

Target symbol					
CHRD	NOG	BMPR1A	BMPR2	BMPR1B	ACVR1
ACVR2A	ACVR2B	BMP7	BMP2	BMP6	GREM1
SMAD4	FST	BMPER	GREM2	GATA4	SHH
NBL1	HJV	MSX1	ACVRL1	FOXF1	FGF10
TBX6	TWSG1	TBXT	SOST	FGF2	FGF8
ACVR1B	FGF4	BGN	WNT3A	SMAD5	RUNX2
LEF1	RGMB	FGF18	MSX2	FGF9	NKX2-5
CTNNB1	SMAD2	TGFBR1	SMAD9	PAX9	AMHR2
BGLAP	FSTL1	NANOG	SOSTDC1	PITX2	TGFBR3
CD34	NEO1	SOX2	POU5F1	FBN1	BARX1
SPP1	FGF13	SOX17	ACVR1C	SMAD6	DLX2
SOX9	MGP	FGF7	DKK1	FN1	PRDM14
GDNF	CCN2	DLX5	WFIKKN1		

Table S14. Targets of FGF9 from STRING database.

**Target symbol**					
FGFR3	FGFR1	FGFR2	FGFR4	KL	EGF
HSPG2	FGFBP1	DCN	KDR	EGFR	FGF23
FLT4	SOX9	KLB	PDGFRB	FRS2	NTRK1
CDH2	SHH	CD44	FLT1	HGF	PDGFRA
MET	IGF1R	KIT	TEK	NRP1	SRY
ERBB2	TGFB1	INSR	BMP4	INS	CSF1R
FLT3	FN1	FGF20	TNFSF12	ERBB4	BMP2
ERBB3	MICU3	FGFRL1	NTRK2	GPC1	IGF1
GRB2	BDNF	NGFR	MICU2	SDC1	CXCL8
FGF19	GATA4	ALB	NOG	PECAM1	MAPK3
EPHA2	DHH	TNFRSF12A	HDGF	IL6	KRAS

Table S15. Predicted targets of the five reprogramming compounds after removing duplicates.

Target symbol					
CCNB3	CDK1	CCNB1	CCNB2	GSK3B	GSK3A
BACE1	SLC29A1	ADORA3	PTGES	CDK6	CCKBR
MAPK1	MAOA	HIF1A	CHEK1	BLM	PYGL
PYGM	GCK	PSEN2	PSENEN	NCSTN	APH1A
PSEN1	APH1B	QPCTL	HCRTR2	HCRTR1	ADAMTS5
CTSS	CSNK1D	PTPN1	TACR3	CDK9	CCNT1
ERBB2	PIK3CD	PRKACA	AKT1	CCNA2	CDK2
FPR1	CALCRL	TTK	IL2	IDH1	PTAFR
TOP2A	ALOX5	TRPV1	TNKS2	ITGAV	ITGB3
MMP9	MMP1	MMP2	SLC2A3	CFD	GRK2
CCNE1	EPHX2	MDM2	GPR119	TAAR1	TRPM8
MAPKAPK2	AGTR1	PDE3A	NTRK1	PDE3B	FPR2
QPCT	FKBP1A	MAP3K8	CYP26A1	CPT1A	PDE4A
PDE4C	CYP19A1	MKNK2	NOS2	TERT	BAD
LIMK2	NAMPT	PDE5A	FLT1	PPIA	MAOB
BDKRB1	RPS6KB1	IDO1	GRPR	F2	CDK7
SCP2	AQP1	KCNJ5	ADH5	AKR1C4	CHIA
ADH1A	ADH4	ADH1B	ADH1C	ADH7	ADH6
GSK-3A	GSK-3B	ERK2	FGF4	PTN	HBEGF
FGFR2	FGF7	KLB	SERPINC1	F10	SELP
FGFR1	PF4	HGF	EGFR	ATIII	SERPIND1
ALOX12B	ACVR1C	ACVR1B	INHBA	TGFBR1	ACVR2B
BMPR1A	ACVR2A	FST	FSTL3	INHBB	INHA
NODAL	IGSF1	SMAD3	LOC102723407	SMAD2	BMPER
BMPR2	BMPR1B	BMP7	BMP2	BMP6	GREM1

GATA4	SHH	NBL1	HJV	MSX1	FOXF1
TBXT	SOST	BGN	WNT3A	SMAD5	RUNX2
FGF18	MSX2	FGF9	NKX2-5	CTNNB1	SMAD9
BGLAP	FSTL1	NANOG	SOSTDC1	PITX2	TGFBR3
SOX2	POU5F1	FBN1	BARX1	SPP1	FGF13
DLX2	SOX9	MGP	DKK1	FN1	PRDM14
DLX5	WFIKKN1	TBX6	FGFR3	KL	EGF
KDR	FGF23	FLT4	PDGFRB	FRS2	CDH2
MET	IGF1R	KIT	TEK	NRP1	SRY
INS	CSF1R	FLT3	FGF20	TNFSF12	ERBB4
FGFRL1	NTRK2	GPC1	IGF1	GRB2	BDNF
SDC1	CXCL8	ALB	PECAM1	MAPK3	EPHA2
HDGF	IL6	KRAS	DHH	TNFRSF12A	MICU2
AURKB	SLC2A1	CD34	NEO1	CCNH	ACHE
AVPR2	AVPR1A	SOX17	SMAD6	KCNJ3	KCNJ4
VDR	NR3C2	GDNF	CCN2	BCL2L12	ENSG00000268643
CNR1	ITK	HSPG2	DCN	FGF2	FGF1
PDE4B	PDE4D	CD44	PDGFRA	FGFR4	FGF19
MAPK11	WEE1	INSR	BMP4	FGF8	FGFBP1
GRM4	STK17B	ERBB3	MICU3	ACVR1	ACVRL1
SMAD4	GREM2	LEF1	RGMB	TGFB3	TGFB1
FGF10	TWSG1	PAX9	AMHR2	CHRD	NOG
NGFR					

Table S16. Characteristics of 109 common targets.

Target symbol	Degree	Betweenness Centrality
ALB	63	0.10274169
FGF2	62	0.03320747
TGFB1	62	0.07321279
BMP4	58	0.0483372
FGF8	57	0.03627999
FGF10	57	0.02021198
FGF4	56	0.02194524
FN1	56	0.03150587
CD44	55	0.02474713
IGF1	54	0.03104927
EGFR	53	0.06866983
BMP2	52	0.03766919
PECAM1	49	0.03258433
SHH	48	0.01887932
KDR	47	0.01992301
HGF	45	0.00877467
SPP1	44	0.01693776
SMAD3	44	0.01251807
FGF13	43	0.0062804
SOX2	42	0.01119066
RUNX2	42	0.01376434
CDH2	40	0.00865495
PDGFRA	39	0.01828181
TGFB3	39	0.00806068
PDGFRB	38	0.00384604
POU5F1	38	0.00590951

MMP2	38	0.02876928
NOG	35	0.00859897
NANOG	34	0.00257592
GATA4	34	0.01295648
DKK1	33	0.00529056
DCN	32	0.00568371
SOX17	30	0.00404458
BMP6	30	0.00966472
BGN	29	0.00296713
TEK	28	0.00361548
LEF1	27	0.00251744
MSX1	26	0.00283532
MMP1	26	0.00311903
NTRK1	26	0.00343416
NRP1	26	0.00183296
FGF19	25	0.00255561
TERT	24	0.0152609
CSF1R	24	0.00271202
MSX2	22	0.0025101
FGFR3	21	0.00184034
ITGB3	21	0.00191402
GDNF	21	0.00033233
ITGAV	21	0.00157431
GREM1	19	0.00264992
NKX2-5	19	0.00059946
NTRK2	19	0.00472648
AGTR1	19	0.03959275
TGFBR3	18	0.00355993

ERBB4	17	0.00018064
FLT3	17	0.00409557
PF4	17	0.00212492
FBN1	17	0.00189747
F2	17	0.03141339
SMAD6	17	0.00173994
ACVRL1	17	0.00233585
FLT4	16	0.00183217
NODAL	16	0.00114635
PITX2	14	0.0012064
SERPINC1	14	0.0016649
FOXF1	13	0.00349166
CCNA2	13	0.02399871
DLX5	12	0.00002225
CTSS	12	0.00138141
MGP	12	0.00161346
SMAD9	12	0.00044863
ACVR1C	12	0.00073042
IDO1	11	0.00043245
PRDM14	10	0.00004916
GREM2	10	0.0002537
BMPER	10	0.00075626
AMHR2	9	0.00120337
BARX1	8	0.00018093
SERPIND1	8	0.00016975
SOSTDC1	7	0.0000527
F10	7	0.00004668
BACE1	7	0.00079538

KLB	6	0
CYP19A1	6	0.0005883
NOS2	6	0.00024336
FSTL3	5	0.00014863
AURKB	5	0.00242924
GRPR	5	0.00207833
BDKRB1	5	0.00212534
WFIKKN1	4	0.00007526
MAPK11	4	0.00020173
PTAFR	4	0.00014153
BLM	3	0.00044317
TTK	3	0.00012277
CFD	3	0
GCK	3	0.00075761
CCKBR	3	0
NR3C2	3	0
TACR3	3	0.0001683
PYGM	2	0.0001589
AQP1	2	0
AVPR1A	2	0
GRM4	1	0
KCNJ3	1	0
ALOX5	1	0
SLC29A1	0	0
PDE4C	0	0
PDE3A	0	0
CALCRL	0	0

Table S17. Original data for Figure 2F.

qPCR	Repeat	M-Day 0	M-Day 7	O-Day 3	O-Day 8	O-Day 13	O-Day 18
OCT4/GAPDH	#1	0.9500	0.0092	0.0015	0.0044	0.0041	0.0040
mRNA levels	#2	1.1100	0.0063	0.0023	0.0064	0.0093	0.0054
	#3	0.9000	0.0060	0.0018	0.0039	0.0093	0.0103
	Mean	0.9867	0.0072	0.0019	0.0049	0.0076	0.0066
	SD	0.1097	0.0018	0.0004	0.0013	0.0030	0.0033
PAX2/GAPDH	#1	0.9100	8.4542	2.8113	0.8874	0.3926	0.3222
mRNA levels	#2	1.1200	6.0316	4.0344	1.5304	0.9329	0.4769
	#3	1.0000	8.9685	5.3458	1.6293	1.1288	0.1237
	Mean	1.0100	7.8181	4.0639	1.3490	0.8181	0.3076
	SD	0.1054	1.5684	1.2675	0.4028	0.3813	0.1771

Table S18. Original data for Figure 2G.

qPCR	Repeat	M-Day 0	M-Day 7	O-Day 3	O-Day 8	O-Day 13	O-Day 18
WT1/GAPDH	#1	0.97	11.46	210.79	884.17	883.34	505.16
mRNA levels	#2	0.92	9.08	247.84	875.32	916.24	798.53
	#3	1.02	9.29	216.93	1291.75	760.21	599.40
	Mean	0.97	9.94	225.19	1017.08	853.26	634.36
	SD	0.05	1.32	19.86	237.91	82.25	149.78
GATA4/GAPDH	#1	1.13	1311.28	2547.83	3350.82	2508.43	970.16
mRNA levels	#2	1.03	577.25	1631.52	1809.99	2762.17	1310.99
	#3	0.85	1291.87	2481.93	3953.64	2737.75	1162.41
	Mean	1.00	1060.13	2220.43	3038.15	2669.45	1147.85
	SD	0.14	418.30	511.07	1105.50	139.98	170.88
NR0B1/GAPDH	#1	0.91	114.32	239.85	164.86	32.40	6.12
mRNA levels	#2	1.05	59.15	318.67	48.21	22.99	7.65
	#3	1.00	62.16	144.74	59.22	26.90	8.96
	Mean	0.99	78.54	234.42	90.76	27.43	7.58
	SD	0.07	31.02	87.09	64.40	4.73	1.42
HSD3B2/GAPDH	#1	1.00	43.69	152.58	85.28	1065.15	425.51
mRNA levels	#2	0.97	74.57	229.48	260.88	493.38	284.03
	#3	1.11	35.43	97.47	211.51	365.18	450.28
	Mean	1.03	51.23	159.84	185.89	641.24	386.60
	SD	0.07	20.63	66.30	90.56	372.67	89.70
ZFPM2/GAPDH	#1	0.94	78.69	100.34	182.44	236.42	120.95
mRNA levels	#2	1.02	68.77	116.51	146.34	286.81	208.30

	#3	1.00	65.66	141.71	126.21	151.42	151.68
	Mean	0.99	71.04	119.52	151.66	224.88	160.31
	SD	0.04	6.81	20.85	28.49	68.43	44.31
EMX2/GAPDH	#1	1.19	11.90	11.97	326.23	443.92	361.50
mRNA levels	#2	0.91	16.90	2.15	236.10	251.44	217.21
	#3	1.00	14.11	7.16	349.43	223.47	170.98
	Mean	1.03	14.31	7.09	303.92	306.28	249.90
	SD	0.14	2.51	4.91	59.87	120.02	99.38

Table S19. Original data for Figure 2H.

qPCR	Repeat	M-Day 0	M-Day 7	O-Day 3	O-Day 8	O-Day 13	O-Day 18
SOX9/GAPDH	#1	1.19	3.91	6.42	8.19	7.52	8.33
mRNA levels	#2	0.96	3.52	5.73	13.36	14.15	12.82
	#3	1.00	3.71	6.57	9.09	8.63	17.12
	Mean	1.05	3.72	6.24	10.21	10.10	12.76
	SD	0.12	0.19	0.45	2.76	3.55	4.39
FGF9/GAPDH	#1	1.00	4.85	3.78	6.39	4.24	8.25
mRNA levels	#2	1.15	2.74	2.63	1.69	3.53	4.01
	#3	0.92	4.89	7.21	6.38	9.06	8.15
	Mean	1.02	4.16	4.54	4.82	5.61	6.80
	SD	0.12	1.23	2.38	2.71	3.01	2.42
CLDN11/GAPDH	#1	1.00	6.85	1.96	36.07	36.19	73.20
mRNA levels	#2	0.94	7.97	1.47	50.04	84.41	137.01
	#3	1.12	2.67	1.55	29.51	49.32	62.95
	Mean	1.02	5.83	1.66	38.54	56.64	91.05
	SD	0.09	2.80	0.26	10.49	24.93	40.13
FSHR/GAPDH	#1	1.10	1435.57	100.48	78.30	21.70	5.77
mRNA levels	#2	1.00	1201.49	92.52	70.48	38.25	7.51
	#3	0.89	910.76	71.60	62.26	61.54	14.55
	Mean	1.00	1182.61	88.20	70.35	40.50	9.28
	SD	0.11	262.91	14.92	8.02	20.02	4.65

Table S20. Original data for Figure 2I.

qPCR	Repeat	M-Day 0	M-Day 7	O-Day 3	O-Day 8	O-Day 13	O-Day 18
HSD3B1/GAPDH	#1	1.15	214.13	545.89	363.94	279.87	127.67
mRNA levels	#2	1.00	246.54	440.38	444.48	292.18	97.86
	#3	0.91	166.00	285.19	441.15	588.43	229.29
	Mean	1.02	208.89	423.82	416.52	386.83	151.60
	SD	0.12	40.52	131.13	45.57	174.70	68.91
CYP17A1/GAPDH	#1	1.00	2.80	6.86	16.14	25.22	32.28
mRNA levels	#2	1.16	1.19	4.14	12.15	13.77	19.69
	#3	1.24	1.14	7.21	17.61	32.28	20.85
	Mean	1.13	1.71	6.07	15.30	23.76	24.28
	SD	0.12	0.94	1.68	2.82	9.34	6.96
HSD17B3/GAPDH	#1	1.00	24.70	8.55	29.07	42.61	26.06
mRNA levels	#2	0.97	30.20	10.84	25.66	43.26	28.81
	#3	1.05	12.79	6.67	68.94	58.85	45.03
	Mean	1.01	22.56	8.69	41.23	48.24	33.30
	SD	0.04	8.90	2.09	24.07	9.19	10.25
